# β‐adrenergic modulation of discrimination learning and memory in the auditory cortex

**DOI:** 10.1111/ejn.14480

**Published:** 2019-07-01

**Authors:** Horst Schicknick, Julia U. Henschke, Eike Budinger, Frank W. Ohl, Eckart D. Gundelfinger, Wolfgang Tischmeyer

**Affiliations:** ^1^ Special Lab Molecular Biological Techniques Leibniz Institute for Neurobiology Magdeburg Germany; ^2^ Department Systems Physiology of Learning Leibniz Institute for Neurobiology Magdeburg Germany; ^3^ Institute of Cognitive Neurology and Dementia Research Otto von Guericke University Magdeburg Magdeburg Germany; ^4^ Center for Behavioral Brain Sciences Magdeburg Germany; ^5^ Institute of Biology Otto von Guericke University Magdeburg Magdeburg Germany; ^6^ Department Neurochemistry and Molecular Biology Leibniz Institute for Neurobiology Magdeburg Germany; ^7^ Molecular Neurobiology Medical Faculty Otto von Guericke University Magdeburg Magdeburg Germany

**Keywords:** acquisition, atenolol, clenbuterol, consolidation, ICI118,551, isoproterenol, Mongolian gerbil, propranolol, reconsolidation, xamoterol

## Abstract

Despite vast literature on catecholaminergic neuromodulation of auditory cortex functioning in general, knowledge about its role for long‐term memory formation is scarce. Our previous pharmacological studies on cortex‐dependent frequency‐modulated tone‐sweep discrimination learning of Mongolian gerbils showed that auditory‐cortical D_1/5_‐dopamine receptor activity facilitates memory consolidation and anterograde memory formation. Considering overlapping functions of D_1/5_‐dopamine receptors and β‐adrenoceptors, we hypothesised a role of β‐adrenergic signalling in the auditory cortex for sweep discrimination learning and memory. Supporting this hypothesis, the β_1/2_‐adrenoceptor antagonist propranolol bilaterally applied to the gerbil auditory cortex after task acquisition prevented the discrimination increment that was normally monitored 1 day later. The increment in the total number of hurdle crossings performed in response to the sweeps per se was normal. Propranolol infusion after the seventh training session suppressed the previously established sweep discrimination. The suppressive effect required antagonist injection in a narrow post‐session time window. When applied to the auditory cortex 1 day before initial conditioning, β_1_‐adrenoceptor‐antagonising and β_1_‐adrenoceptor‐stimulating agents retarded and facilitated, respectively, sweep discrimination learning, whereas β_2_‐selective drugs were ineffective. In contrast, single‐sweep detection learning was normal after propranolol infusion. By immunohistochemistry, β_1_‐ and β_2_‐adrenoceptors were identified on the neuropil and somata of pyramidal and non‐pyramidal neurons of the gerbil auditory cortex. The present findings suggest that β‐adrenergic signalling in the auditory cortex has task‐related importance for discrimination learning of complex sounds: as previously shown for D_1/5_‐dopamine receptor signalling, β‐adrenoceptor activity supports long‐term memory consolidation and reconsolidation; additionally, tonic input through β_1_‐adrenoceptors may control mechanisms permissive for memory acquisition.

## Graphical abstract

β‐adrenergic signalling in the auditory cortex controls mechanisms that permit cortex‐dependent frequency‐modulated tone‐sweep discrimination learning and support memory retention and/or retrieval after learning and rehearsal.

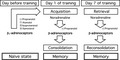

**Abbreviations**
ANOVA: analysis of variance
AR: avoidance rate
cAMP: cyclic adenosine monophosphate
CR+: correct conditioned response
CR: conditioned response
CR−: false alarm
CS: conditioned stimulus
*D*: discrimination rate
FM: frequency‐modulated tone
NMDA: N‐methyl‐D‐aspartate
RM‐ANOVA: repeated‐measures analysis of variance

## 1 INTRODUCTION

Post‐acquisition consolidation processes are relevant for the stabilisation and subsequent retrieval of long‐term memory (Dudai, Karni, & Born, 2015; Izquierdo et al., 2006; Kandel, Dudai, & Mayford, 2014; Korte & Schmitz, 2016; Matthies, 1989; McGaugh, 2000; Morris, 2006; Poo et al., 2016; Sara, 2017; Squire, Genzel, Wixted, & Morris, 2015). Reconsolidation processes may be required for the stabilisation and the involvement of new aspects after retrieval of a previously established memory trace (Bonin & De Koninck, 2015; Dudai, 2012; Miranda & Bekinschtein, 2018; Nader, 2015; Roesler, 2017; Sara, 2010; Tronson & Taylor, 2007). Both consolidation and reconsolidation processes are sensitive to interfering treatments within limited time windows (Drumond, Madeira, & Fonseca, 2017; McGaugh & Roozendaal, 2009).

The catecholamine neuromodulators noradrenaline (norepinephrine) and dopamine are known to facilitate plasticity mechanisms required for memory consolidation and reconsolidation (Alberini & Ledoux, 2013; Bouret & Sara, 2005; Diergaarde, Schoffelmeer, & De Vries, 2008; Hagena, Hansen, & Manahan‐Vaughan, 2016; Hansen, 2017; Hansen & Manahan‐Vaughan, 2014; Moncada, 2017; Moncada, Ballarini, Martinez, Frey, & Viola, 2011; Roozendaal & McGaugh, 2011; Sara, 2015, 2017; Yamasaki & Takeuchi, 2017). Noradrenaline is released in multiple brain regions from terminals of locus coeruleus neurons (Aston‐Jones & Cohen, 2005; Atzori et al., 2016; Gu, 2002; Levitt & Moore, 1978; Rho, Kim, & Lee, 2018). Coeruleo‐cortical terminals may also be a source of dopamine release (Devoto & Flore, 2006; McNamara & Dupret, 2017). Noradrenaline and dopamine exert differential modulatory effects on excitatory and inhibitory neurotransmission (Mather, Clewett, Sakaki, & Harley, 2016; Salgado, Trevino, & Atzori, 2016; Xing, Li, & Gao, 2016) and—through mediation of β‐adrenergic and D_1/5_‐dopaminergic receptors, respectively—play permissive roles in activity‐dependent and N‐methyl‐D‐aspartate (NMDA)‐type glutamate receptor‐gated modifications of neuronal connections (Andrzejewski, McKee, Baldwin, Burns, & Hernandez, 2013; Bavelier, Levi, Li, Dan, & Hensch, 2010; Castner & Williams, 2007; O'Dell, Connor, Guglietta, & Nguyen, 2015).

The mammalian auditory cortex participates in particular aspects of auditory stimulus processing, task‐specific forms of auditory performance, and learning and memory (Aitkin, 1990; Angeloni & Geffen, 2018; Ehret, 1997; Gaucher et al., 2013; Grosso, Cambiaghi, Concina, Sacco, & Sacchetti, 2015; Kuchibhotla & Bathellier, 2018; Ohl, 2015; Scheich et al., 2011; Weinberger, 2015). Learning alters the neural processing of sounds. When a sound acquires a special meaning by learning, its neuronal representation in the auditory cortex changes. Different types of representational rearrangements may occur, with distinct functional importance for the cognitive operations (*e.g*., detection, discrimination) performed with relevant cues in a given type of learning task as well as for associative features of a novel sound required to become behaviourally meaningful (Ohl, 2015; Scheich et al., 2011; Weinberger, 2015). Learning‐induced plasticity in the auditory cortex associates with long‐term consolidation and structural changes in synaptic connections (Froemke & Schreiner, 2015; Galvan & Weinberger, 2002; Kraus et al., 2002; Moczulska et al., 2013).

Multiple neuromodulatory systems, including cholinergic, serotonergic, dopaminergic and noradrenergic signalling, play in part complementary roles in the regulation of information processing and plastic rearrangements in the auditory cortex (Atzori, Kanold, Pineda, Flores‐Hernandez, & Paz, 2005; Edeline, 2012; Froemke & Martins, 2011; Jacob & Nienborg, 2018; Scheich et al., 2011; Thiel, 2007; Weinberger, 2015). The cortical release of dopamine and noradrenaline was shown to increase in response to tones paired with reinforcement (Feenstra, Vogel, Botterblom, Joosten, & de Bruin, 2001; Mingote, de Bruin, & Feenstra, 2004). Noradrenaline is necessary for experience‐dependent plasticity in the developing (Shepard, Liles, Weinshenker, & Liu, 2015) and adult auditory cortex (Edeline, Manunta, & Hennevin, 2011; Glennon et al., 2019; Manunta & Edeline, 2004; Martins & Froemke, 2015). Moreover, noradrenergic input facilitates the discriminative abilities of cortical neurons (Devilbiss, 2019; Gaucher & Edeline, 2015; Manunta & Edeline, 1997, 1999; Shakhawat et al., 2015) and enhances the establishment of stimulus‐reinforcement contingencies during initial stages of discriminative learning (Bouret & Sara, 2005; Janitzky et al., 2015).

The auditory cortex is crucial for associative learning mediated through detection, discrimination and categorisation of frequency‐modulated tone (FM) sweeps (Letzkus et al., 2011; Ohl, Scheich, & Freeman, 2001; Ohl, Wetzel, Wagner, Rech, & Scheich, 1999; Rybalko, Suta, Nwabueze‐Ogbo, & Syka, 2006; Wetzel, Ohl, & Scheich, 2008). During and shortly after conditioning of Mongolian gerbils to FMs in a shuttle‐box, increased dopamine responses were monitored in auditory‐ and prefrontal‐cortical regions, which may indicate the acquisition of appropriate representational changes and relevant associations (Stark, Rothe, Wagner, & Scheich, 2004; Stark & Scheich, 1997) and promote the readout of task‐related information (Deliano et al., 2018; Happel, Deliano, Handschuh, & Ohl, 2014). Indeed, catecholaminergic modulation may comprise local circuits within the auditory cortex and feedback connections with multiple brain regions (Budinger & Scheich, 2009; Happel, 2016; Henschke, Noesselt, Scheich, & Budinger, 2015; Homma et al., 2017; Jarvers et al., 2016; Schulz, Woldeit, Goncalves, Saldeitis, & Ohl, 2016) that are thought to integrate auditory stimulus processing with non‐auditory cognitive functions, thus enabling meaningful associations and behavioural response selection (Grosso et al., 2015; Ohl, 2015; Scheich et al., 2011; Shamma & Fritz, 2014; Weinberger, 2015).

Despite a vast amount of work on catecholaminergic neuromodulation of auditory cortex functioning, knowledge about the role of auditory‐cortical catecholamine signalling for long‐term memory formation is still rather limited. We previously have shown that, in Mongolian gerbils, memory consolidation in the FM sweep discrimination paradigm requires activation of NMDA‐type glutamate receptors, the protein kinase mTOR and protein synthesis in the auditory cortex (Kraus et al., 2002; Schicknick & Tischmeyer, 2006; Tischmeyer et al., 2003) and that dopamine is likely to participate in the regulation of mechanisms that are involved in the formation of persistent memory. Specifically, inhibition of auditory‐cortical D_1/5_‐dopamine receptors within a limited time window shortly after conditioning impaired the retention of newly acquired memory (Schicknick et al., 2012), whereas artificial pharmacological activation of this class of dopamine receptors shortly after or even 1 day before initial conditioning induced mTOR‐mediated, protein synthesis‐dependent changes in the gerbil brain that persist for >24 hr and facilitate memory consolidation (Reichenbach et al., 2015; Schicknick et al., 2008), but not the acquisition performance. Updating a previously acquired memory trace by additional training is subject to dopaminergic modulation as well (Rothe, Deliano, Scheich, & Stark, 2009; Schicknick et al., 2012).

Catecholamines individually and complementarily modulate cortical functions (Chandler, Waterhouse, & Gao, 2014). As recently shown for several learning tasks involving hippocampal and cortical brain regions, dopamine signalling by D_1/5_‐receptors and noradrenaline signalling by β‐receptors may independently modulate memory consolidation (Cavalcante et al., 2017; Moncada, 2017; Moncada et al., 2011; Ouyang, Young, Lestini, Schutsky, & Thomas, 2012). Based on these considerations, we hypothesised a functional importance also of β‐adrenergic signalling in the auditory cortex for auditory discrimination learning and memory. To test this hypothesis, β‐adrenergic antagonists and agonists were bilaterally applied to the auditory cortex of gerbils, and the effects of pharmacological treatments on learning and memory were examined during behavioural training in a shuttle‐box performed on subsequent days. A scheme of the temporal relations between pharmacological treatments and behavioural training sessions is given in Figure S1. (a) To address a potential role of β‐adrenergic signalling for the consolidation of newly acquired FM discrimination memory, the effects of the β_1/2_‐adrenoceptor antagonist propranolol locally applied shortly after the first session of differential conditioning were examined in Experiment 1. (b) Aiming at processes potentially relevant for the reconsolidation of reactivated, previously established FM discrimination memory, the effects of propranolol applied after training of already repeatedly trained gerbils were tested in Experiments 2 and 3. (c) Given that pharmacological dopamine receptor activation facilitates anterograde memory formation (Schicknick et al., 2008) and that interference with central noradrenergic signalling in naive animals, that is, without explicit stimulation or task engagement, may cause long‐lasting impacts on synaptic transmission and on learning and memory in discriminative paradigms (Church, Flexner, Flexner, & Rainbow, 1985; Everitt, Robbins, Gaskin, & Fray, 1983; Flexner, Church, Flexner, & Rainbow, 1984; Flexner, Flexner, Church, Rainbow, & Brunswick, 1985; Kemp & Manahan‐Vaughan, 2008), we examined in Experiments 4–7 if and how β‐adrenoceptor antagonists and agonists influence FM discrimination learning and memory when administered one day prior to initial training. (d) Finally, in Experiment 8 we studied if and how propranolol administered one day prior to initial training influences learning in a simpler auditory learning paradigm, that is, FM detection‐conditioned active avoidance learning in the shuttle‐box.

## 2 MATERIALS AND METHODS

### 2.1 Animals

One hundred thirty‐one male 3‐month‐old Mongolian gerbils (*Meriones unguiculatus*) were used. The animals were housed in groups of five and given free access to standard laboratory chow and tap water on a 12‐hr light/dark cycle (light on at 6 a.m.). Animal experimentation was approved by the animal care committee of the Land Sachsen‐Anhalt in accordance with the regulations of the German Federal Law on the Care and Use of Laboratory Animals and with the Council Directive 2010/63EU of the European Parliament and the Council of 22 September 2010 on the protection of animals used for scientific purposes.

### 2.2 Pharmacological agents

(S)‐(‐)‐Propranolol hydrochloride [classified as mixed β_1_‐ and β_2_‐adrenergic antagonists (Hoffmann, Leitz, Oberdorf‐Maass, Lohse, & Klotz, 2004)], atenolol [classified as selective β_1_‐adrenergic antagonist (Hoffmann et al., 2004), but see (Baker, 2005)], ICI118,551 hydrochloride [classified as selective β_2_‐adrenergic antagonist (Hoffmann et al., 2004)], (‐)‐isoproterenol bitartrate [classified as mixed β_1_‐, β_2_‐ and β_3_‐adrenergic agonist (Baker, 2010; Hoffmann et al., 2004)] and clenbuterol hydrochloride [classified as selective β_2_‐adrenergic agonist (Baker, 2010)] were obtained from Sigma‐Aldrich (Germany). Xamoterol hemifumarate [classified as β_1_‐adrenoceptor‐selective partial agonist (Ardestani et al., 2017)] was obtained from Tocris Bioscience (UK). For intracortical injections of 1 μl portions (see below), drugs were dissolved in 0.9% saline to the following concentrations: propranolol, 10 μg/μl = 33.8 nmol/μl = 33.8 mM; atenolol, 1 μg/μl = 3.8 nmol/μl = 3.8 mM; ICI118,551, 0.1 ng/μl = 0.32 pmol/μl = 0.32 μM; isoproterenol, 1 μg/μl = 2.8 nmol/μl = 2.8 mM; clenbuterol, 0.1 μg/μl = 0.32 nmol/μl = 0.32 mM; and xamoterol, 4 μg/μl = 10 nmol/μl = 10 mM. The doses used were previously shown to affect learning and memory or to reverse memory‐enhancing agonist effects after intracerebral injection in rats (Bahar, Samuel, Hazvi, & Dudai, 2003; Berman, Hazvi, Neduva, & Dudai, 2000; Ferry, Roozendaal, & McGaugh, 1999; Lennartz, Hellems, Mook, & Gold, 1996; Ramos, Colgan, Nou, & Arnsten, 2008; Roozendaal & Cools, 1994).

### 2.3 Surgical procedures and intracortical injections

Surgery and intracortical injections were performed as described in detail in our previous studies (Budinger, Heil, & Scheich, 2000; Kraus et al., 2002; Schicknick et al., 2012). In brief, on the day before intracortical injections, gerbils were deeply anesthetised by intraperitoneal injection of 4 mg ketamine and 3 mg xylazine per 100 g body weight, the cranial skin was disinfected and incised, and three holes of about 1 mm in diameter were drilled into the left and right side of the skull at locations above the primary, anterior and posterior fields of the auditory cortex (Radtke‐Schuller et al., 2016). After surgery, gerbils were allowed to recover for 1 day before injections were performed. At different times in relation to behavioural training (see scheme of pharmacological treatments in Figure S1), 1 μl portions of drug solution or vehicle (0.9% saline) were applied per target region under light halothane anaesthesia (1.5%–2% halothane in air for respiration) over a period of 4 min. The timing of noradrenergic pharmacological interference in relation to the behavioural experiments is based on our previous studies on dopaminergic mechanisms of FM discrimination in the gerbil auditory cortex (Schicknick et al., 2008, 2012) and on studies on sensory discrimination in rats (Eschenko & Sara, 2008; Guzman‐Ramos, Osorio‐Gomez, Moreno‐Castilla, & Bermudez‐Rattoni, 2012; Tronel, Feenstra, & Sara, 2004). A double, 2‐hr spaced injection of pharmacological agents was chosen because intracerebrally applied propranolol is eliminated with a half‐life of ≈2 hr (Smits & Struyker‐Boudier, 1979), while cortical noradrenaline signalling >2 hr after behavioural training might also be of relevance to memory processing (Tronel et al., 2004). To allow for temporal precision, the lengths of the intervals between the end of training and the time of injections were varied (Experiments 2 and 3). The positioning of the injection tracks has previously been validated (Kraus et al., 2002). The described procedure of local drug delivery, including surgery, intracortical injection and anaesthesia, caused no impairments in FM discrimination learning and performance (see methodological considerations in the Supporting Information).

### 2.4 Behavioural experiments

Schematic figures depicting the sequence of events, stimulus durations, stimulation pauses, trial and intertrial interval durations described in the behavioural protocols below are given in Figure S2.

#### 2.4.1 FM discrimination paradigm

Gerbils were trained once per day in a two‐way shuttle‐box to discriminate the modulation direction of linearly modulated FM sweeps in a Go/NoGo procedure as described earlier (Kraus et al., 2002; Schicknick et al., 2012). Briefly, before each training session, gerbils were allowed to habituate for 3 min to the training chamber without acoustical stimulation and foot shock. During the sessions, the animals were trained to discriminate between conditioned stimuli (CSs) consisting of sequences (250‐ms tone, 250‐ms pause) of either an ascending (1–2 kHz, CS+) or a descending FM (2–1 kHz, CS‐). A training session consisted of 60 trials, that is, 30 presentations of each CS+ and CS‐ in a pseudo‐randomized order, and lasted ≈25 min. The mean intertrial interval was 15 s. To avoid mild foot shocks, gerbils had to cross a hurdle within 6 s of CS+ presentation and to suppress this response within 6 s of CS‐ presentation. Hurdle crossings within 6 s upon the onset of CS+ and CS‐ were regarded as correct conditioned responses (CR+) and false alarms (CR−), respectively. For each experiment, the numbers of CR+ and CR− were monitored per session as documented in the Figures S4–S10. To quantify the discrimination performance, the relative frequencies of CR+ and CR− were calculated as percentage of trials with presentations of CS+ and CS‐, respectively. Subsequently, the discrimination rate *D*, that is, the difference between the relative frequencies of CR+ and CR−, was calculated. To assess treatment effects on learning of the hurdle crossings in response to the sweeps per se, the relative frequency of conditioned responses (∑CR), that is, the sum of CR+ and CR− expressed as per cent of the total number of trials, was calculated. To assess treatment effects on arousal and activity, the numbers of hurdle crossings during the habituation period preceding each session as well as the intertrial activity, that is, the numbers of hurdle crossings occurring between the trials of each session, were monitored. To assess treatment effects on sensory, motivational and motor systems, the avoidance latencies, that is, the times required to change the compartment during CR+, and the number of escape reactions, that is, hurdle crossings in response to the foot shock, were recorded within the sessions. For each experiment, these data are documented in Figures S4–S10.

#### 2.4.2 FM detection paradigm

In this two‐way active avoidance experiment, gerbils were trained once per day in the shuttle‐box to detect a single FM sweep. Before each training session, gerbils were allowed to habituate for 3 min to the training chamber without acoustical stimulation and foot shock. During the sessions, the animals were trained to a CS consisting of sequences (250‐ms tone, 250‐ms pause) of an ascending FM (1–2 kHz). A training session consisted of 60 trials and lasted ≈25 min. The mean intertrial interval was 15 s. To avoid mild foot shocks, gerbils had to cross the hurdle within 6 s of CS presentation. A hurdle crossing within 6 s upon the onset of the CS was regarded as conditioned response (CR). The avoidance rate (AR) was calculated as the percentage of trials with CR relative to the total number of trials. The numbers of hurdle crossings during the habituation period preceding each session, as well as the numbers of escape reactions and intertrial crossings, and the avoidance latencies, that is, the times required to change the compartment during CR, monitored per session are documented in Figure S11.

### 2.5 Statistics

All behavioural data are presented as group means ± *SEM*. Data without index or linked to the index “session” were expressed as group means per session. When linked to the index “block”, a training session was subdivided into five blocks of trials (*cf*. Figure S2A), and data were expressed as group means per trial block. Each trial block consisted of 12 consecutive trials, that is, six presentations of each CS+ and CS‐. For statistical evaluation, StatView 5.0.1 (SAS) was used. Analysis of variance (ANOVA) and repeated‐measures analysis of variance (RM‐ANOVA, with training session and/or trial block serving as the repeated measures) were performed as indicated. Fisher's protected least significant difference (PLSD) or Dunnett's test was used for *post hoc* comparisons. Student's two‐tailed *t* tests for paired or unpaired comparisons were used where appropriate. *p* Values of <0.05 were considered as statistically significant.

### 2.6 Immunohistochemistry

Gerbils (*n *=* *3) were deeply anesthetised by intraperitoneal injection of 20 mg ketamine and 3 mg xylazine per 100 g body weight and perfused transcardially with 20 ml of 0.1 M phosphate‐buffered saline (PBS, pH 7.4) followed by 200 ml of 4% paraformaldehyde. The brains were removed, post‐fixed overnight in 4% paraformaldehyde at 4°C and then cryoprotected by soaking them in 30% sucrose in PBS for 48 hr. Brains were cut on a cryostat (Leica CM 1950, Germany) into 50‐μm‐thick horizontal sections. Sections were collected in PBS, washed, blocked and then incubated in either anti‐ β_1_ receptor antibody solution (Santa Cruz Biotechnologies, USA, ab sc‐568, 1:500) or anti‐ β_2_ receptor antibody solution (Abcam, USA, AB 13989; 1:1,000) overnight. Then, the antibody reaction was visualized using the avidin‐biotin method (ABC kit, Vectors Laboratories) and diaminobenzidine as the chromogen (Sigma‐Aldrich, Germany, D8001). Every sixth section was counterstained for cytoarchitecture using cresyl violet (Nissl‐stain). After washing and dehydrating, sections were then mounted on gelatine‐coated slides and coverslipped with Merckoglas (Merck, Germany).

## 3 RESULTS

### 3.1 Effect of propranolol applied after initial FM discrimination training

Previous studies on the dopamine system demonstrated that auditory‐cortical catecholaminergic neuromodulation after acquisition of the FM discrimination supports memory consolidation (Schicknick et al., 2008, 2012). Experiment 1 of the present study was performed to test for effects of auditory‐cortical β‐adrenoceptor antagonism after the initial learning event on the FM discrimination in subsequent sessions. A scheme of the pharmacological treatment is given in Figure S1. Gerbils were trained on the FM discrimination task for three sessions. The β‐adrenoceptor antagonist propranolol was applied to the auditory cortex twice, that is, immediately after and 2 hr after completion of the first training session. Gerbils of the control group received vehicle.

**Figure 1:**
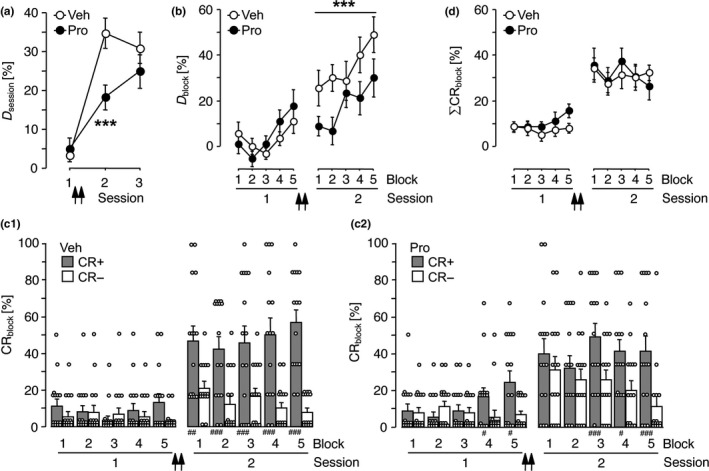
Post‐acquisition propranolol application caused a discrimination deficit in session 2. Data were collected in Experiment 1. Gerbils were trained on the FM discrimination (1–2 kHz vs. 2–1 kHz) for 3 sessions. Injections of vehicle (Veh, *n *=* *15) or 33.8 mM propranolol (Pro, *n *=* *15) were applied to the auditory cortex twice, that is, immediately after and 2 hr after completion of session 1. (a) Discrimination rates per training session, that is, the differences between the relative frequencies of correct conditioned responses (CR+) and false alarms (CR‐) per training session, are referred to as *D*_session_ [%] in the *y*‐axis label (group means ± *SEM*). (b–d) Each of sessions 1 and 2 was subdivided into 5 blocks of 12 trials (*cf*. Figure S2A). (b) Discrimination rates per trial block, that is, the differences between the relative frequencies of CR+ and CR‐ per trial block, are referred to as *D*_block_ [%] in the *y*‐axis label (group means ± *SEM*). (c_1–2_) Relative frequencies of CR+ (filled bars) and CR‐ (empty bars) per trial block are referred to as CR_block_ [%] in the *y*‐axis label for the Veh (c_1_) and Pro (c_2_) group (individual data points and group means + *SEM*). (d) Total frequencies of hurdle crossings per trial block, that is, the sums of the relative frequencies of CR+ and CR‐ per trial block, are referred to as ∑CR_block_ [%] in the *y*‐axis label (group means ± *SEM*). Arrows indicate the approximate injection times. ***p <* *0.005, significant group difference (a: *t* test; b: RM‐ANOVA). ^#^ *p *<* *0.05, ^##^ *p *<* *0.01, ^###^ *p *<* *0.005, significantly different from the corresponding CR‐ rate (*t* test)

Figure 1A shows the mean discrimination rates calculated per training session (*D*_session_). RM‐ANOVA comparing *D*_session_ over sessions across treatment groups revealed a significant effect of session (*F*_2,56_ = 41.14, *p *<* *0.0001) and a significant session x treatment interaction (*F*_2,56_ = 4.89, *p *=* *0.011). The effect of treatment missed statistical significance (*F*_1,28_ = 4.03, *p *=* *0.054). The session effect is indicative of an improvement of the discrimination performance over sessions; the session x treatment interaction indicates differential effects of the pharmacological treatments on the slopes of the learning curves. To assess the origin of this interaction, *D*_session_ was analysed separately within pharmacological treatment groups and within training sessions. A significant session effect was evident within each treatment group (vehicle: *F*_2,28_ = 30.65, *p *<* *0.0001; propranolol: *F*_2,28_ = 13.38, *p *<* *0.0001), indicating that both groups improved their discrimination scores over sessions. Within‐session comparison revealed no significant group difference in session 1 (*t*_28_ = 0.59, *p * = * *0.558), demonstrating that both experimental groups showed comparable acquisition performances during the initial training, that is, before pharmacological treatment. In session 2, one day after injections, *D*_session_ of the propranolol‐treated group was significantly lower than that of controls (*t*_28_ = 3.25, *p *=* *0.003). In session 3, the group difference in *D*_session_ was no longer statistically significant (*t*_28_ = 1.02, *p *=* *0.319). Thus, as measured by the discrimination rates calculated as an average per training session, propranolol locally infused into the auditory cortex after the initial training affected mainly the increment in discrimination performance that was normally detectable in session 2.

To elucidate whether post‐acquisition propranolol infusion had effects on retention and retrieval of memory already acquired during session 1 or on performance gains during session 2, data collected in training sessions 1 and 2 of Experiment 1 were subdivided into five trial blocks per session (*cf*. Figure S2A). The mean discrimination rates per trial block (*D*_block_) in antagonist‐treated gerbils and controls are illustrated in Figure 1B. RM‐ANOVA comparing *D*_block_ over sessions and trial blocks across treatment groups revealed significant effects of session (*F*_1,28_ = 57.99, *p *<* *0.0001) and treatment (*F*_1,28_ = 6.26, *p *=* *0.018) and a significant session x treatment interaction (*F*_1,28_ = 10.08, *p *=* *0.004), thus confirming the impact of propranolol on the discrimination increment over sessions 1–2 already disclosed above. Moreover, the effect of trial block was statistically significant (*F*_4,112_ = 7.45, *p *<* *0.0001), whereas, importantly, interactions of the factor trial block with the factors session and treatment were not evident (block × session: *F*_4,112_ = 0.73, *p *=* *0.569; block × treatment: *F*_4,112_ = 0.87, *p *=* *0.482; block × session × treatment: *F*_4,112_ = 0.40, *p *=* *0.807). This indicates that both experimental groups were similarly able to improve the discrimination performance and to form a short‐term memory of it within the pre‐injection session 1 and within the post‐injection session 2. Consistently, analysis of *D*_block_ within session 2 revealed significant effects of treatment (*F*_1,28_ = 10.86, *p *=* *0.003) and block (*F*_4,112_ = 3.49, *p *=* *0.01) but no block x treatment interaction (*F*_4,112_ = 0.46, *p *=* *0.763). These results suggest that post‐acquisition propranolol infusions affect the retention, retrieval and/or behavioural manifestation of memory relevant for FM discrimination that was already acquired in the initial training session, but not performance gains during session 2.

To further assess the effect of post‐acquisition propranolol injection, the differences in *D*_block_ between trial block 1 of session 2 and trial block 5 of session 1 were calculated. These values differed significantly between groups (*t*_28_ = 2.19, *p *=* *0.037), exhibiting positive values for controls (14.44 ± 7.43%) but negative values for propranolol‐treated gerbils (−8.89 ± 7.61%). This indicates that on average the discrimination performance of controls improved whereas that of propranolol‐treated gerbils declined during the training‐free intersession interval.

For a more detailed examination, the rates of CR+ and CR− were compared within each trial block of sessions 1 and 2. Both experimental groups performed on average more CR+ than CR− in trial blocks 4 and 5 of session 1, that is, prior to pharmacological treatment (Figure 1C_1_,C_2_). For the propranolol group, this increase in CR+ versus CR− reached even statistical significance. Nevertheless, this group performed nearly as many CR− as CR+ in trial blocks 1 and 2 of session 2, that is, at the day after propranolol treatment. Over the course of session 2, propranolol‐treated gerbils relearned the discrimination; in trial blocks 3, 4 and 5, they performed significantly more CR+ than CR−. In contrast, the control group performed significantly more CR+ than CR− throughout session 2, starting already with trial block 1.

The discrimination deficit of propranolol‐treated gerbils in trial blocks 1 and 2 of session 2 was due to non‐significant inverse changes in the rates of CR+ and CR− compared to vehicle controls. Consequently, as demonstrated in Figure 1D, the relative frequencies of total hurdle crossings in response to the FMs per se (∑CR_block_, calculated from the sum of CR+ and CR− per trial block) were very similar and showed a similar increase over sessions for propranolol‐treated gerbils and controls. RM‐ANOVA comparing ∑CR_block_ over sessions and trial blocks across treatment groups revealed a significant session effect (*F*_1,28_ = 56.26, *p *<* *0.0001), but no significant effects of treatment (*F*_1,28_ = 0.36, *p *=* *0.554) and trial block (*F*_4,112_ = 0.59, *p *=* *0.672), and no significant interactions (session x treatment: *F*_1,28_ = 0.15, *p *=* *0.700; block × treatment: *F*_4,112_ = 0.25, *p *=* *0.911; session × block: *F*_4,112_ = 1.52, *p *=* *0.202; session × block × treatment: *F*_4,112_ = 0.98, *p *=* *0.424). Thus, propranolol‐treated gerbils learned and performed the hurdle reactions in response to the sounds per se normally.

To look for side effects of propranolol on arousal and activity and on sensory, motivational and/or motor mechanisms that may interfere with learning and memory performance on subsequent days, general parameters, such as the intertrial activities, avoidance latencies and numbers of escape reactions performed during the training sessions were recorded (Figure S4). Compared to vehicle‐treated controls, RM‐ANOVA revealed no significant changes in any of these parameters. As propranolol‐treated gerbils tended to show higher intertrial activity than controls during session 2, linear regression analysis was performed to assess interactions between the intertrial activity and the FM discrimination rate. It revealed no significant correlation (|*R*| = 0.013, *F*_1,28_ = 0.004, *p *=* *0.947).

To summarise, post‐acquisition propranolol infusion into the auditory cortex impaired processes normally occurring during the training‐free interval between sessions 1 and 2 that are relevant for retention and/or retrieval of the newly acquired FM discrimination memory and its further improvement.

### 3.2 Effect of propranolol applied after FM discrimination retraining

The following experiments were performed to assess potential effects of post‐session β‐adrenergic blockade in the auditory cortex of already repeatedly trained gerbils on processes involved in retention, retrieval, reconsolidation and expression of the previously learned FM discrimination. Gerbils were trained for 10 sessions. Surgery was performed after session 6, and propranolol was injected locally into the auditory cortex after session 7. The effect of propranolol treatment compared with the pre‐injection performance level was tested in subsequent training sessions. Two independent experiments were performed to examine whether propranolol treatment within different post‐session time windows exerts differential effects. Schemes of the pharmacological treatments are given in Figure S1.

**Figure 2:**
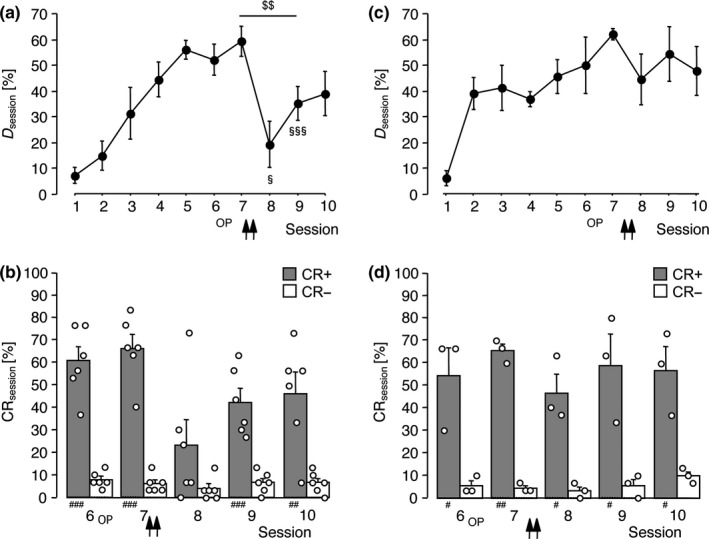
Immediate but not delayed propranolol application after the 7th training session suppressed the previously established FM discrimination. Data were collected in Experiments 2 (a, b; *n *=* *6) and 3 (c, d; *n *=* *3). Gerbils were trained on the FM discrimination for 10 sessions. Surgical operation (OP) was performed after session 6. Propranolol (33.8 mM) was applied to the auditory cortex twice, that is, immediately and 2 hr (a, b) or 2 and 4 hr (c, d) after completion of session 7. (a, c) Discrimination rates per training session (group means ± *SEM*). (b, d) Relative frequencies of correct conditioned responses (CR+) and false alarms (CR‐) per session for training sessions 6–10 (individual data points and group means + *SEM*). Arrows indicate the approximate injection times. ^$$^ *p *<* *0.01, significant session effect (RM‐ANOVA). ^§^ *p *<* *0.05, ^§§§^ *p *<* *0.005, significantly different from the value in session 7 (*t* test). ^#^ *p *<* *0.05, ^##^ *p *<* *0.01, ^###^ *p *<* *0.005, significantly different from the corresponding CR‐ rate (*t* test). Note, gerbils that received propranolol immediately after session 7 failed to discriminate between CS+ and CS‐ in session 8 (b)

In Experiment 2, propranolol was applied twice, that is, immediately and 2 hr after completion of session 7. The mean discrimination rates are shown in Figure 2A. RM‐ANOVA comparing *D*_session_ over sessions 7–9 revealed a significant effect of session (*F*_2,10_ = 7.74, *p *=* *0.009). Comparison with the pre‐injection performance in session 7 showed that propranolol infused immediately after session 7 significantly impaired the discrimination in session 8 (*t*_5_ = 3.12, *p *=* *0.026) and, to a lesser extent, in session 9 (*t*_5_ = 5.30, *p *=* *0.003). In session 10, the difference in *D*_session_ compared to session 7 was no longer statistically significant (*t*_5_ = 2.05, *p *=* *0.095). Comparing the rates of CR+ and CR− showed that the ability to discriminate between the FMs was severely impaired in session 8 but was re‐acquired during subsequent training (Figure 2B). As shown in Figure S5, the propranolol‐induced discrimination deficit was due to a significant decrease in the CR+ rate compared to session 7 (session 8: *t*_5_ = 2.77, *p *=* *0.040; session 9: *t*_5_ = 5.03, *p *=* *0.004). Simultaneously, the number of escape reactions tended to increase, implying that these gerbils were sufficiently motivated by the foot shock. Other parameters recorded during Experiment 2 were not significantly affected.

In Experiment 3, propranolol was infused with a delay of 2 hr compared to Experiment 2, that is, at 2 and 4 hr after completion of session 7. Figure 2C shows the mean discrimination rates per session. RM‐ANOVA comparing *D*_session_ over sessions 7–9 revealed no significant effect of session (*F*_2,4_ = 2.31, *p *=* *0.215). Accordingly, comparison of the CR+ and CR− rates showed that the ability to discriminate the FMs was not compromised after delayed propranolol treatment (Figure 2D). Similarly, analysis of other behavioural parameters recorded during Experiment 3 revealed no significant changes (Figure S6).

To summarise, propranolol infused into the auditory cortex after the 7th training session suppressed the previously established FM discrimination on the subsequent day. The suppressive action was reversible and required antagonist treatment within a narrow post‐session time window.

### 3.3 Effects of β‐adrenoceptor antagonists and agonists applied 1 day before initial FM discrimination training

A temporary β‐blockade may cause long‐lasting alterations in synaptic transmission and discriminative memory processing (Flexner et al., 1985; Kemp & Manahan‐Vaughan, 2008). In Experiments 4–6, we therefore examined the sensitivity of FM discrimination learning to β‐adrenergic antagonists applied to the auditory cortex of untrained gerbils 1 day prior to the start of the behavioural experiments. Given that artificial dopamine receptor activation facilitates anterograde memory formation (Schicknick et al., 2008), β‐adrenergic agonists were tested in Experiment 7 utilising the same injection schedule. A scheme of the pharmacological treatments is given in Figure S1.

**Figure 3:**
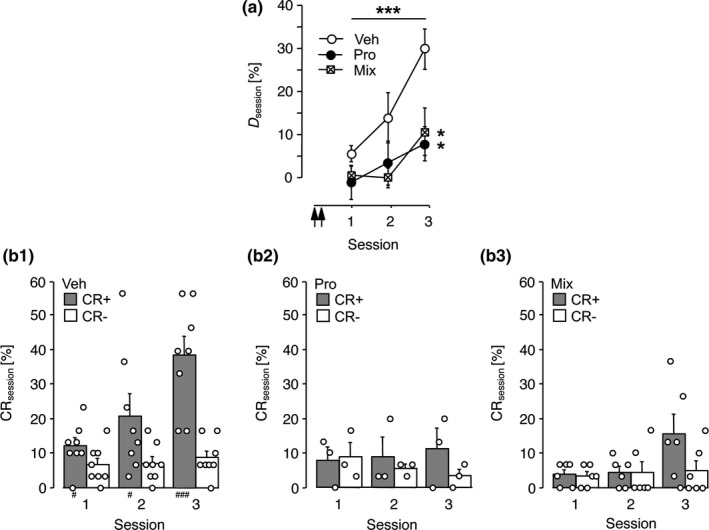
Inhibition of auditory‐cortical β‐adrenoceptors 1 day before conditioning impaired FM discrimination learning. Data were collected in Experiment 4. Gerbils were trained on the FM discrimination for three sessions. Vehicle (Veh, *n *=* *8), 33.8 mM propranolol (Pro, *n *=* *3) or a mixture (Mix, *n *=* *6) containing 3.8 mM atenolol and 0.32 μM ICI118,551 was applied to the auditory cortex twice, that is, 24 and 22 hr prior to the beginning of session 1. (a) Discrimination rates per training session expressed as group means ± *SEM*; arrows indicate the approximate injection times. (b_1–3_) Relative frequencies of correct conditioned responses (CR+) and false alarms (CR‐) per session for gerbils of the Veh (b_1_), Pro (b_2_) and Mix group (b_3_) are shown as individual data points and group means + *SEM*. ***p <* *0.005, significant treatment effect (RM‐ANOVA). *p <* *0.05, significantly different from Veh (Dunnett's test). ^#^ *p *<* *0.05, ^###^ *p *<* *0.005, significantly different from the corresponding CR‐ rate (*t* test). Note, both groups of antagonist‐treated gerbils failed to discriminate between CS+ and CS‐

To assess long‐term effects of an auditory‐cortical β‐adrenoceptor blockade on FM discrimination learning, the gerbils of Experiment 4 received injections of propranolol or vehicle into the auditory cortex twice, that is, 24 and 22 hr before the first training session started. To control for β‐adrenoceptor‐independent drug effects in the context of this experiment, we tested in another group how a mixture of atenolol and ICI118,551 (classified as β_1_‐ and β_2_‐selective antagonists, respectively) influences FM discrimination learning. The effects of the pharmacological treatments were examined in three training sessions. Figure 3A shows the mean discrimination rates per session. RM‐ANOVA comparing *D*_session_ over sessions across treatment groups revealed significant effects of session (*F*_2,28_ = 6.38, *p *=* *0.005) and treatment (*F*_2,14_ = 10.55, *p *=* *0.002) and no treatment x session interaction (*F*_4,28_ = 0.99, *p *=* *0.428). *Post hoc* analysis showed that the discrimination performance of both the propranolol‐treated group and the mixed antagonist‐treated group significantly differed from the performance of vehicle‐treated controls (*p *<* *0.05, Dunnett's test). Accordingly, while the control group achieved significantly higher rates of CR+ than CR− (Figure 3B_1_), neither the propranolol‐treated group (Figure 3B_2_) nor the mixed antagonist‐treated group (Figure 3B_3_) was able to significantly discriminate between the FMs within the scope of Experiment 4. Thus, the β_1,2_‐adrenoceptor antagonist propranolol as well as a mixture of subtype‐selective antagonists locally applied to the auditory cortex of untrained gerbils similarly impaired FM discrimination learning on subsequent days.

**Figure 4:**
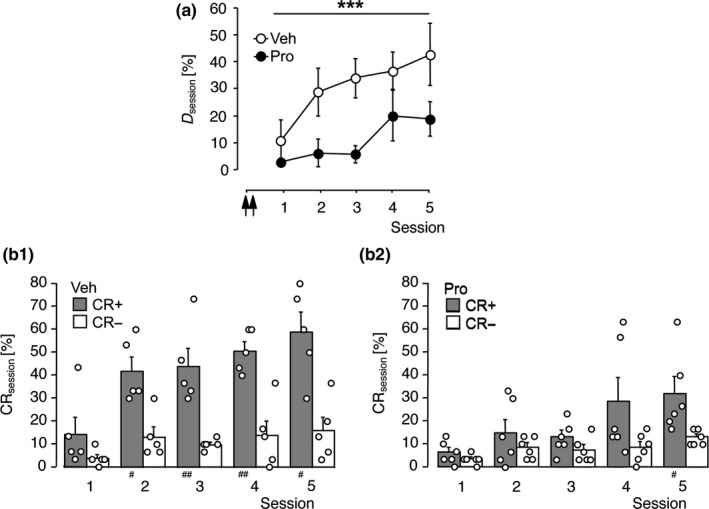
Extended training enabled FM discrimination learning after propranolol treatment. Data were collected in Experiment 5. Gerbils were trained on the FM discrimination for five sessions. Vehicle (Veh, *n *=* *5) or 33.8 mM propranolol (Pro, *n *=* *6) was applied to the auditory cortex twice, that is, 24 and 22 hr prior to the start of session 1. (a) Discrimination rates per training session expressed as group means ± *SEM*; arrows indicate the approximate injection times. (b_1–2_) Relative frequencies of correct conditioned responses (CR+) and false alarms (CR‐) per session for gerbils of the Veh (b_1_) and Pro‐group (b_2_) are shown as individual data points and group means + *SEM*. ***p <* *0.005, significant treatment effect (RM‐ANOVA). ^#^ *p *<* *0.05, ^##^ *p *<* *0.01, significantly different from the corresponding CR‐ rate (*t* test). Note, the Pro‐group achieved a significant difference between the rates of CR+ and CR‐ in session 5

Experiment 5 addressed the persistency of the suppressive effect of auditory‐cortical β‐adrenoceptor blockade on FM discrimination learning. Gerbils were infused with propranolol or vehicle and subsequently trained as in Experiment 4, except that the number of training sessions was raised to 5. The mean discrimination rates are shown in Figure 4A. RM‐ANOVA comparing *D*_session_ over sessions 1–5 across treatment groups revealed a significant antagonist‐induced discrimination deficit (treatment effect: *F*_1,9_ = 14.86, *p *=* *0.004; session effect: *F*_4,36_ = 3.88, *p *=* *0.010; treatment x session: *F*_4,36_ = 0.66, *p *=* *0.623), thus confirming the findings of Experiment 4. Accordingly, vehicle‐treated controls significantly increased the CR+ rate over the CR− rate already during the initial three training sessions (Figure 4B_1_), while the propranolol‐treated gerbils failed (Figure 4B_2_). However, with the extension to five training sessions also the propranolol‐treated group was able to significantly discriminate between FMs.

In both Experiments 4 and 5, the rate of CR+ was significantly compromised by the antagonists compared to vehicle controls (Figures S7 and S8). This effect was accompanied by higher numbers of escape reactions, suggesting that pre‐training infusion of β‐blockers does not cause motivational or motor deficits. Other parameters recorded during the training sessions were not significantly affected.

**Figure 5:**
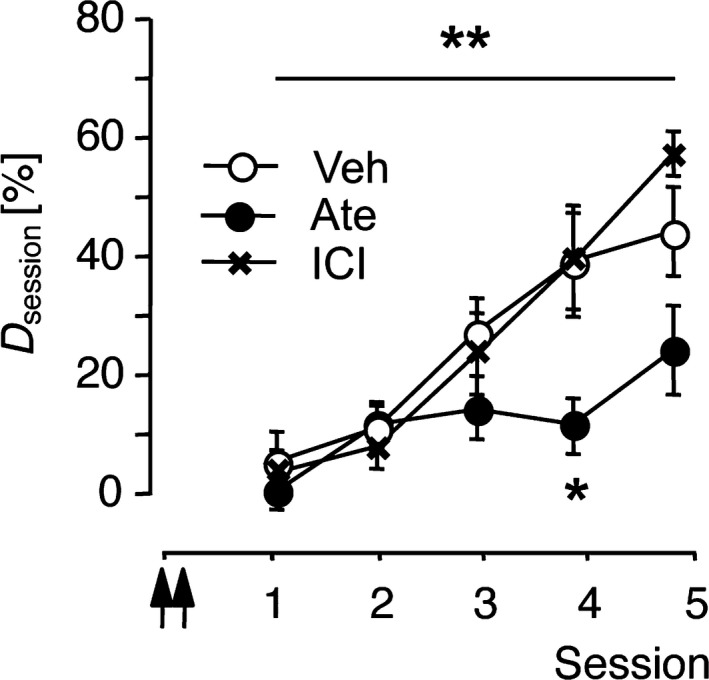
Subtype‐selective β_1_‐adrenoceptor inhibition 1 day before initial conditioning impaired FM discrimination learning. Data were collected in Experiment 6. Gerbils were trained on the FM discrimination for five sessions. Vehicle (Veh, *n *=* *9), 3.8 mM atenolol (Ate, *n *=* *6) or 0.32 μM ICI118,551 (ICI,* n *=* *3) was applied to the auditory cortex twice, that is, 24 and 22 hr prior to the start of session 1. Discrimination rates per training session are shown as group means ± *SEM*. Arrows indicate the approximate injection times. **p <* *0.01, significant treatment x session interaction (RM‐ANOVA). *p <* *0.05, significantly different from Veh (Dunnett's test)

Experiment 6 was performed to assess receptor subtypes concerned with the retarding effect of β‐blockers on FM discrimination learning. Gerbils were trained on the FM discrimination for five sessions. Vehicle, atenolol or ICI118,551 was infused in the auditory cortex twice, that is, 24 and 22 hr prior to the start of the first training session. RM‐ANOVA comparing *D*_session_ over sessions 1–5 across treatment groups revealed no significant effect of treatment (*F*_2,15_ = 2.76, *p *=* *0.095), a significant effect of session (*F*_4,60_ = 29.35, *p *<* *0.0001), and, importantly, a significant treatment x session interaction (*F*_8,60_ = 2.93, *p *=* *0.008). Figure 5 displays differential effects of the pharmacological treatments on the slopes of the learning curves, with a slower increase in *D*_session_ for atenolol‐treated gerbils. Within‐group RM‐ANOVA over sessions 1–5 showed that all three experimental groups significantly improved their discrimination rates (session effect: vehicle, *F*_4,32_ = 16.07, *p *<* *0.0001; atenolol, *F*_4,20_ = 5.67, *p *=* *0.003; ICI118,551, *F*_4,8_=27.74, *p *<* *0.0001). However, within‐session analyses across treatment groups confirmed that atenolol‐treated gerbils achieved lower levels of *D*_session_ than vehicle‐treated controls; this effect reached statistical significance in session 4 (ANOVA: *F*_2,15_=3.86, *p *=* *0.045; *post hoc* Dunnett's test: *p *<* *0.05). Consistent with Experiments 4 and 5, the retardation of FM discrimination learning in the atenolol‐treated group was due to a significantly compromised CR+ rate compared to controls (Figure S9). Other behavioural parameters recorded during the training sessions were not significantly different between the treatment groups.

In Experiments 4–6, temporary inhibition of β‐adrenergic receptors in the gerbil auditory cortex one day prior to initial conditioning considerably retarded FM discrimination learning, implying a tonic effect of β‐adrenergic input. Considering facilitating effects of pharmacological dopamine receptor activation on anterograde memory formation (Schicknick et al., 2008), we examined in Experiment 7 whether the prior application of β‐adrenergic agonists improves FM discrimination learning. For this purpose, the subtype non‐selective β‐adrenergic receptor agonist isoproterenol or vehicle was infused in the auditory cortex of gerbils twice, that is, 24 and 22 hr prior to the first differential conditioning to FMs. Moreover, while the results of Experiment 6 suggest a predominant importance of auditory‐cortical β_1_‐adrenoceptors for FM discrimination learning, previous studies on the role of prefrontal‐cortical adrenoceptors for working memory performance pointed to the β_2_‐subtype and implied that cortical β_1_‐ and β_2_‐adrenoceptors may even play opposing roles in learning and memory processes (Ramos et al., 2005, 2008). In two additional groups of Experiment 7, we tested therefore how xamoterol and clenbuterol, classified as β_1_‐ and β_2_‐selective agonists, respectively, influence FM discrimination learning. The effects of the pharmacological treatments were examined in three training sessions.

**Figure 6:**
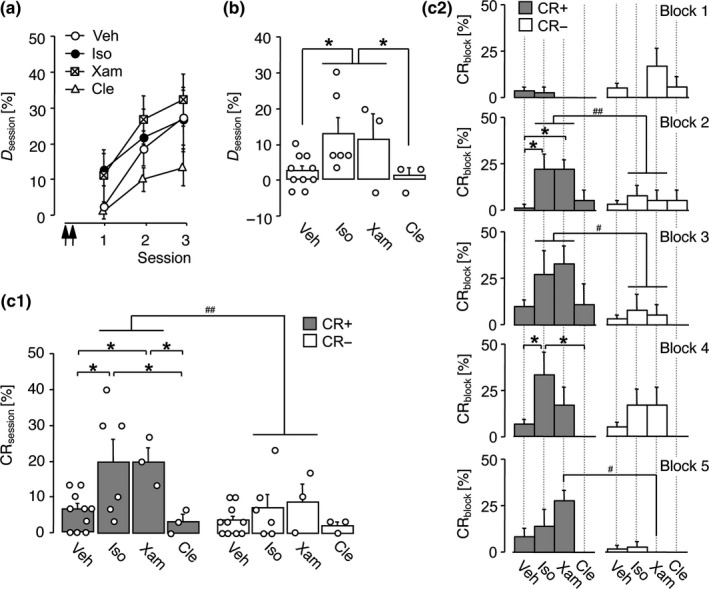
Subtype‐selective β_1_‐adrenoceptor stimulation 1 day before initial conditioning improved FM discrimination learning. Data were collected in Experiment 7. Gerbils were trained on the FM discrimination for three sessions. Vehicle (Veh, *n *=* *10), 2.8 mM isoproterenol (Iso, *n *=* *6), 10 mM xamoterol (Xam, *n *=* *3) or 0.32 mM clenbuterol (Cle, *n *=* *3) was applied to the auditory cortex twice, that is, 24 and  22 hr prior to the start of session 1. (a) Discrimination rates per session are shown for training sessions 1–3 as group means ± *SEM*; arrows indicate the approximate injection times. (b) Discrimination rates per session are shown in detail for training session 1 (individual data points and group means + *SEM*). (c_1–2_) Relative frequencies of correct conditioned responses (CR+) and false alarms (CR‐) in training session 1 are shown, calculated as an average per session (c_1_; individual data points and group means + *SEM*) and per trial block (c_2_; group means + *SEM*). *p <* *0.05, significant group difference (Fisher's PLSD). ^#^ *p *<* *0.05, ^##^ *p *<* *0.01, significant difference between the rates of CR+ and CR‐ (*t* test)

The mean discrimination rates per training session are shown in Figure 6A. RM‐ANOVA comparing *D*_session_ over sessions 1–3 across treatment groups revealed a significant effect of session (*F*_2,36_ = 8.96, *p *=* *0.001) but neither a significant effect of treatment (*F*_3,18_ = 0.99, *p *=* *0.419) nor a treatment × session interaction (*F*_6,36_ = 0.35, *p *=* *0.904). Similarly, RM‐ANOVA over training sessions 1–3 did not disclose significant impacts of pharmacological treatments on the rates of CR+ and CR− and on other behavioural parameters recorded in Experiment 7 (Figure S10). Thus, administration of β‐adrenergic agonists one day prior to conditioning seems not to influence the overall capacity of FM discrimination learning.

To assess potential agonist effects on the initial acquisition of the discrimination, the performance within the first training session was analysed. As shown in Figure 6B, gerbils infused with isoproterenol or xamoterol reached, on average, higher discrimination rates in session 1 than clenbuterol‐treated gerbils or vehicle‐treated controls. The effect of treatment, however, did not reach statistical significance (ANOVA: *F*_3,18_ = 2.95, *p *=* *0.060).

Figure 6C_1_ depicts the relative frequencies of CR+ and CR− calculated as an average of session 1. ANOVA across pharmacological treatment conditions revealed a significant effect of treatment on the rate of CR+ (*F*_3,18_ = 4.59, *p *=* *0.015), while group differences in the rate of CR− were not evident (*F*_3,18_ = 1.02, *p *=* *0.406). *Post hoc* analysis showed that gerbils infused with isoproterenol or xamoterol reached significantly higher CR+ rates than vehicle controls or clenbuterol‐treated gerbils (isoproterenol vs. vehicle: *p *=* *0.008; xamoterol vs. vehicle: *p *=* *0.032; isoproterenol vs. clenbuterol: *p *=* *0.019; xamoterol vs. clenbuterol: *p *=* *0.039; Fisher's PLSD). In contrast, the β_2_‐agonist clenbuterol did not significantly affect the CR+ rate compared to vehicle‐treated controls (*p *=* *0.663; Fisher's PLSD).

The performance scores monitored in session 1 for the isoproterenol‐ and xamoterol‐treated groups, that is, gerbils that received either a non‐selective or a selective β_1_‐stimulating compound, were very similar (CR+: *p *>* *0.999; CR−: *p *=* *0.706; *D*:* p *=* *0.770; Fisher's PLSD). Combining their data for further analysis revealed that the composite group consisting of isoproterenol‐ and xamoterol‐treated gerbils reached significantly higher discrimination rates in session 1 than clenbuterol‐treated gerbils and vehicle‐treated controls (Figure 6B; ANOVA: effect of treatment, *F*_2,19_ = 4.61, *p *=* *0.023; *post hoc* Fisher's PLSD: composite group vs. clenbuterol, *p *=* *0.045; composite group vs. vehicle, *p *=* *0.012). Accordingly, the CR+ rate of the composite group significantly exceeded the CR− rate in session 1 (Figure 6C_1_; *t*_8_ = 3.39, *p *=* *0.009; *t* test) whereas clenbuterol‐treated gerbils and vehicle‐treated controls did not acquire a significant FM discrimination within this session (clenbuterol: *t*_2_ = 0.50, *p *=* *0.667; vehicle: *t*_9_ = 1.65, *p *=* *0.132; *t* test).

For a more detailed examination of the agonist effect, session 1 was subdivided into 5 blocks of 12 trials (*cf*. Figure S2A). Figure 6C_2_ shows the rates of CR+ and CR− calculated per trial block of session 1 for each treatment group. In trial block 1, ANOVA did not reveal significant effects of treatment (CR+: *F*_3,18_ = 0.39, *p *=* *0.762; CR−: *F*_3,18_ = 2.52, *p *=* *0.090). This suggests that the agonists did not cause effects on general mechanisms that may interfere with the shuttle‐box performance, such as the sensitivities to the conditioned stimuli or the probability of response initiation. An increase in the CR+ rates of isoproterenol‐ and xamoterol‐treated gerbils over the values of vehicle‐treated controls was first evident in trial block 2 (ANOVA: *F*_3,18_ = 4.72, *p *=* *0.013; Fisher's PLSD: isoproterenol vs. vehicle, *p *=* *0.004; xamoterol vs. vehicle, *p *=* *0.019), while the CR− rate was not affected (ANOVA: *F*_3,18_ = 0.317, *p *=* *0.813). Accordingly, the composite group of isoproterenol‐ and xamoterol‐treated gerbils started to perform significantly more CR+ than CR− in trial block 2 of session 1 (*t*_8_ = 3.41, *p *=* *0.009; *t* test). Thus, in contrast to the clenbuterol‐ or vehicle‐treated groups, those gerbils infused with isoproterenol or xamoterol acquired the discrimination already during the course of the first session of differential conditioning to FMs.

To summarise, when applied to the auditory cortex of gerbils one day prior to the first conditioning session, antagonists and agonists acting at β_1_‐adrenoceptors retard and facilitate, respectively, the initial acquisition of the FM discrimination while β_2_‐adrenoceptor‐selective antagonists and agonists were ineffective.

### 3.4 Effect of propranolol applied 1 day before initial FM detection training

**Figure 7:**
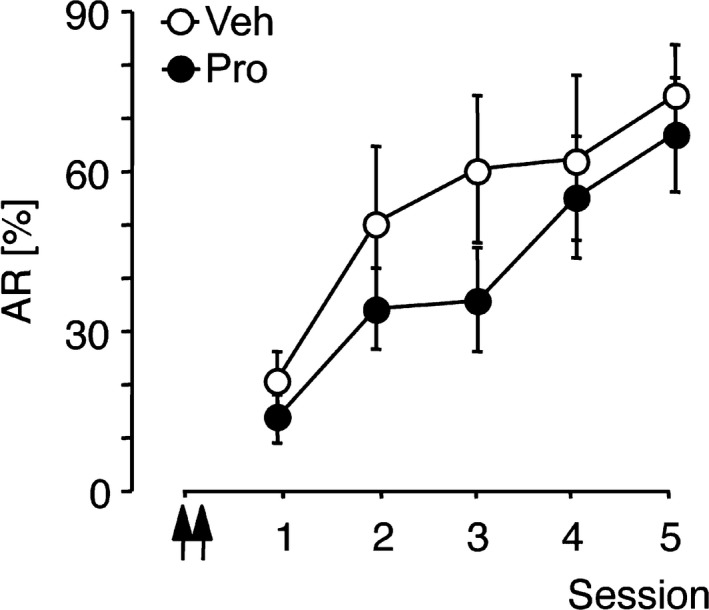
FM detection learning was not affected after auditory‐cortical propranolol infusion. Data were collected in Experiment 8. Gerbils were trained on the two‐way active avoidance task for five sessions to detect a single FM (1–2 kHz). Vehicle (Veh, *n *=* *6) or 33.8 mM propranolol (Pro, *n *=* *6) was applied to the auditory cortex twice, that is, 24 and 22 hr prior to the start of session 1. Avoidance rates per training session are shown. Arrows indicate the approximate injection times. All data points represent group means ± *SEM*

Experiment 8 was performed to assess whether interference with auditory‐cortical β‐adrenergic modulation also affects learning of a simpler auditory task. Gerbils were trained on the FM‐conditioned active avoidance task in the shuttle‐box for 5 sessions. Propranolol or vehicle was infused into the auditory cortex twice, that is, 24 and 22 hr prior to the first training session. Figure 7 shows the mean avoidance rates per training session (AR). RM‐ANOVA comparing AR over sessions across treatment groups revealed a significant effect of session (*F*_4,40_ = 17.42, *p *<* *0.0001), no significant effect of treatment (*F*_1,10_ = 0.97, *p *=* *0.347) and no significant session x treatment interaction (*F*_4,40_ = 0.67, *p *=* *0.618). Similarly, analysis of other behavioural parameters recorded during Experiment 7 revealed no significant changes (Figure S11). Thus, a temporary inhibition of β‐adrenergic receptors in the auditory cortex of untrained gerbils does not significantly affect FM detection‐conditioned active avoidance learning on subsequent days.

### 3.5 Distribution of β_1_‐ and β_2_‐adrenergic receptors in the gerbil auditory cortex

**Figure 8:**
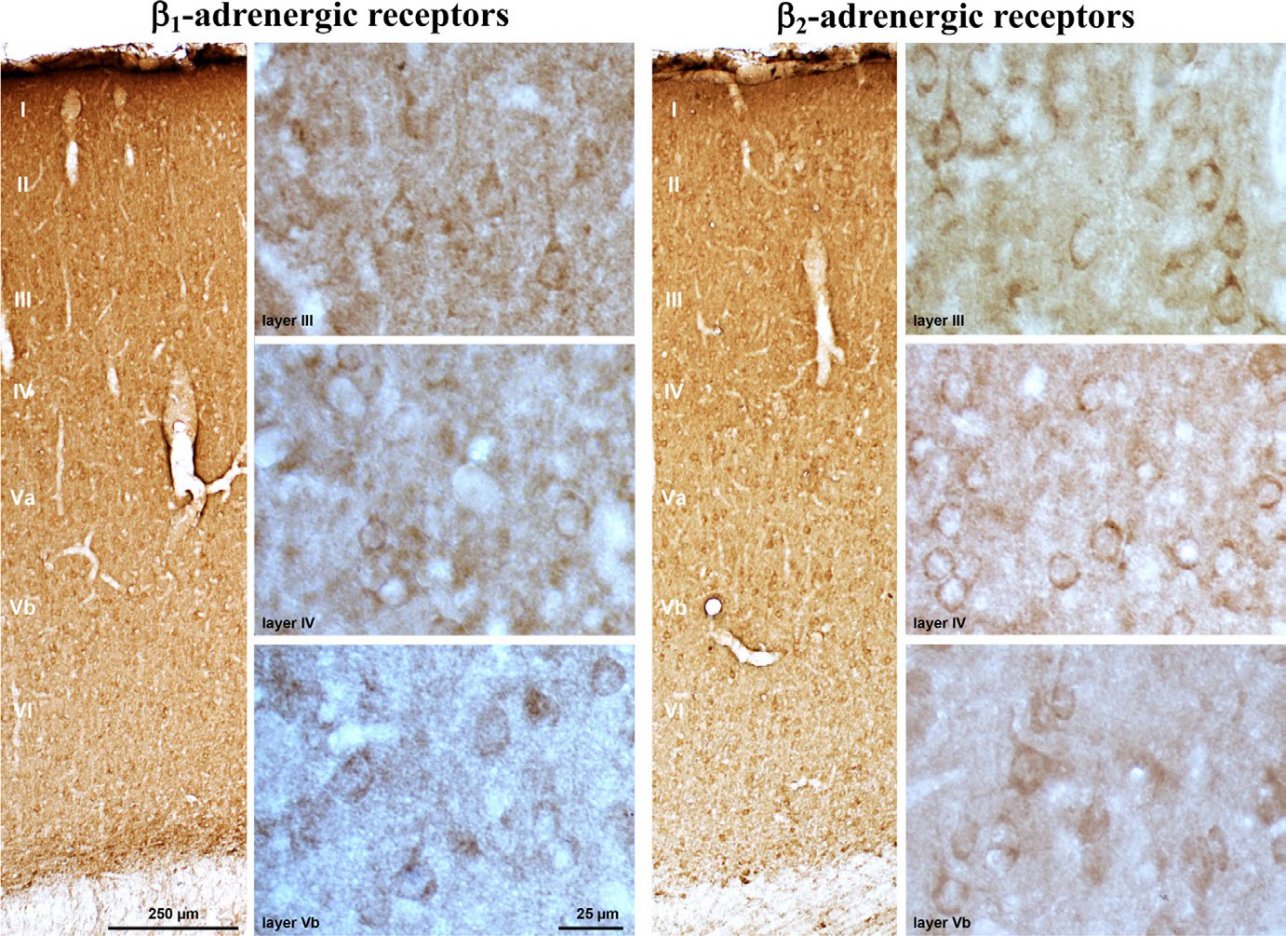
β_1_‐ and β_2_‐adrenergic receptors are abundant in the auditory cortex of the Mongolian gerbil. They can be found widely distributed within the neuropil and at the somata of pyramidal (layers II‐III and V‐VI) and non‐pyramidal neurons (mainly layer IV). [Colour figure can be viewed at http://wileyonlinelibrary.com]

As shown in Figure 8, there was a strong antibody staining against β_1_‐ and β_2_‐adrenergic receptors in the auditory cortex of the Mongolian gerbil, demonstrating the abundance of these receptors within cortical areas also of this species. β_1_‐ and β_2_‐positive puncta within the neuropil most likely represent synapses. The dense staining around the somata of pyramidal (layers II‐III and V‐VI) and non‐pyramidal neurons (mainly layer IV) is consistent with a considerable influence of β‐adrenergic afferents on most types of auditory‐cortical neurons.

## 4 DISCUSSION

This study addressed the functional relevance of β‐adrenoceptor signalling in the gerbil auditory cortex for FM discrimination learning and memory. The β_1/2_‐adrenoceptor antagonist propranolol locally applied shortly after task acquisition impaired the discrimination 1 day later compared to vehicle controls. Post‐session propranolol administration to already repeatedly trained gerbils suppressed the previously established FM discrimination. Delayed injection was ineffective. When applied 1 day prior to initial conditioning, propranolol or a mixture of the β_1_‐selective antagonist atenolol and the β_2_‐selective antagonist ICI118,551, or atenolol (not ICI118,551) alone retarded FM discrimination learning, while the β_1/2/3_‐adrenoceptor agonist isoproterenol and the β_1_‐selective agonist xamoterol (not the β_2_‐selective agonist clenbuterol) facilitated task acquisition. FM detection‐conditioned active avoidance learning was normal after propranolol infusion.

### 4.1 Effect of propranolol applied after initial FM discrimination training

Direct input from the locus coeruleus (Budinger, Laszcz, Lison, Scheich, & Ohl, 2008) and abundant expression of β_1_‐ and β_2_‐adrenergic receptors (Figure 8) indicate that the auditory cortex of the Mongolian gerbil is subject to β‐adrenergic modulation. In rats, transient increases in noradrenergic activity were found for more than 2 hr upon sensory discrimination learning (Eschenko & Sara, 2008; Guzman‐Ramos et al., 2012; Tronel et al., 2004), implying an involvement in memory consolidation. In Experiment 1, blockade of β‐adrenoceptors by propranolol locally applied to the gerbil auditory cortex within 2 hr after initial differential conditioning to FMs impaired the discrimination in the next training session, performed 1 day later. The post‐training injection schedule precluded drug effects on acquisition of the discrimination during initial conditioning. Moreover, propranolol‐treated gerbils started session 2 with a severe discrimination deficit, but during the course of this session the gains in their discrimination rates resembled those of controls. This in turn implies that propranolol applied after the initial conditioning impairs the ability to retain already acquired task‐related memory during the post‐injection intersession interval or to store it in an accessible way. Indeed, the discrimination rate of the antagonist‐treated group decreased on average between the last trial block of session 1 and the first trial block of session 2, while that of the control group increased. These findings strongly suggest that propranolol applied to the auditory cortex during the post‐acquisition phase antagonises actions of endogenous noradrenaline released in response to learning to enable memory storage and improvement over time, that is, memory consolidation (McGaugh, 2000).

For learning an auditory discrimination task, plastic cortical rearrangements have to be induced and subsequently consolidated. We postulate that this is associated with increased contrast formation between neighbouring neurons, thus enabling a more sensitive discrimination of the conditioned stimuli, and with an integration of auditory stimulus processing with non‐auditory cognitive functions, thus enabling meaningful associations and appropriate behavioural response selection. In Experiment 1, the propranolol‐induced discrimination deficit in session 2 was characterised, on average, by a lower CR+ rate and a higher CR− rate compared to controls. The resulting similar response frequencies to CS+ and CS‐ imply that post‐acquisition blockade of auditory‐cortical β‐adrenoceptors affects selectively the consolidation of those aspects of cortical reorganisation that support sound discrimination, while changes required for an association between sound presentation and the likelihood of foot shock reinforcement are retained. Comparable stimulus‐generalising effects were monitored in our previous studies on memory consolidation after post‐acquisition inhibition of auditory‐cortical protein synthesis (Kraus et al., 2002) or of D_1/5_‐dopamine receptors (Schicknick et al., 2012) that are known to be involved in the translational control of FM discrimination memory via the protein kinase mTOR (Schicknick et al., 2008). Both noradrenaline and dopamine, through activation of β‐adrenergic and D_1/5_‐dopaminergic receptors, respectively, may control mTOR‐mediated mechanisms to enhance protein synthesis‐dependent neural plasticity (Beckley et al., 2016; Connor, Wang, & Nguyen, 2011; Gelinas et al., 2007; Lenz & Avruch, 2005; Maity, Rah, Sonenberg, Gkogkas, & Nguyen, 2015). Auditory‐cortical activity of mTOR‐mediated pathways shortly after FM discrimination learning, in turn, is required for long‐lasting memory persistence (Tischmeyer et al., 2003). Accordingly, an involvement of mTOR and translational control mechanisms in learning of FM sound‐driven behaviour has recently been shown in avian species (Ahmadiantehrani & London, 2017; Batista, Johnson, Dominguez, Costa‐Mattioli, & Pena, 2016, 2018). Thus, in conjunction with previous studies, the findings of the present Experiment 1 suggest that dopaminergic and noradrenergic systems in the auditory cortex may interact in the control of memory consolidation required for distinguishing acoustic features of complex sounds and/or their behavioural meanings.

### 4.2 Effect of propranolol applied after FM discrimination retraining

Studies on the dopamine system suggest that updating and stabilisation of reactivated, previously established FM discrimination memory are subject to modulation by cortical catecholamines (Rothe et al., 2009; Schicknick et al., 2012). In the present study, local propranolol infusion shortly after retraining decreased the discrimination performance during subsequent retraining, while the antagonist was ineffective when applied with a delay of 2 hr. This effective period is consistent with studies on olfactory discrimination in rats demonstrating increased locus coeruleus activity within 2 hr upon retrieval from remote memory (Eschenko & Sara, 2008). Assuming similar conditions for auditory discrimination in the gerbil, the present results suggest that post‐session pharmacological interference with noradrenergic signalling in the auditory cortex was only effective in a narrow time window, in which increased endogenous adrenergic activity may contribute to the stabilisation of reactivated memory.

Previous studies on FM discrimination (Kraus et al., 2002; Schicknick & Tischmeyer, 2006; Tischmeyer et al., 2003) and auditory fear (Moczulska et al., 2013) revealed that retention of auditory memories after retrieval may not rely on the same auditory cortex‐mediated mechanisms that are recruited during processing of newly acquired memory. Consistently, in the present Experiments 1 and 2 distinct behavioural effects of post‐session propranolol treatment were recorded. After initial conditioning, the antagonist affected the rates of both CR+ and CR− in opposite directions, while after retraining merely the response to Go‐stimulus presentation was compromised. These findings imply that, depending on the learning history, post‐session auditory‐cortical β‐adrenoceptor activities participate in different mechanisms of memory processing, that is, stabilisation of discriminative memory after initial learning and of goal‐directed aspects upon retraining. Both mechanisms may ultimately be supportive for selecting and pursuing appropriate behavioural response strategies.

### 4.3 Effects of β adrenoceptor antagonists and agonists applied 1 day before initial FM discrimination training

When applied to the auditory cortex of untrained gerbils, propranolol or a mixture of atenolol and ICI118,551, or atenolol (not ICI118,551) alone impaired FM discrimination learning on subsequent days. Propranolol and atenolol are β‐blocking compounds with different physiochemical, pharmacological and pharmacokinetic properties (Gleiter & Deckert, 1996). The finding that they both retard FM discrimination learning increases the confidence that the observed effect was due to their common action at β_1_‐adrenoceptors. Because the firing modes of locus coeruleus neurons during basal states of brain activity may be inappropriate for β‐adrenoceptor activation (Aston‐Jones & Cohen, 2005; Atzori et al., 2016), the induction of the antagonist effect in naive gerbils may have involved drug actions on non‐stimulated receptor functioning. Indeed, β‐adrenoceptors are linked to the cyclic adenosine monophosphate (cAMP) signal transduction system even in the absence of catecholamines (Engelhardt, Grimmer, Fan, & Lohse, 2001), and propranolol, atenolol and ICI118,551 act as inverse agonists towards this pathway (Hoffmann et al., 2004). Therefore, their binding to the receptor may block both transmitter‐induced as well as constitutive cAMP accumulation.

The distinctive impact of β_1_‐adrenoceptor ligands on FM discrimination learning presumably reflects differences in the distribution and/or functional involvement of cortical β_1_‐ and β_2_‐adrenoceptors (Dienel & Cruz, 2016; Grzelka, Kurowski, Gawlak, & Szulczyk, 2017; Gu, 2002; Hein, 2006; Liu, Liang, Ren, & Li, 2014; Rainbow, Parsons, & Wolfe, 1984). In addition, β_1_‐ligands (not ICI118,551) increase constitutive cAMP levels locally via type‐4 cyclic nucleotide phosphodiesterase translocation (Richter, Mika, Blanchard, Day, & Conti, 2013). This action might have contributed to the effects of β_1_‐adrenoceptor ligands on FM discrimination learning in the present study. However, agonists and antagonists of β_1_‐adrenoceptors influenced FM discrimination learning in opposite directions. This can hardly be explained alone with phosphodiesterase translocation or with non‐specific, indirect effects of the artificial ligands. Rather, the facilitating effect of the agonists lends support for the conclusion that the retarding effect of the antagonists was specifically mediated by interference with endogenous β_1_‐receptor functions. The data can be explained by postulating that a temporary artificial activation of auditory‐cortical β_1_‐adrenoceptors of untrained gerbils induces cerebral changes that persist at least until the next day and facilitate FM discrimination learning, so that these animals are enabled to acquire the discrimination already during the initial two trial blocks of the first training session. Conversely, a temporary inhibition of auditory‐cortical β_1_‐adrenergic signalling prior to initial training may cause lasting cerebral changes that considerably retard FM discrimination learning, so that it takes several sessions of differential conditioning until these gerbils are capable to discriminate between the conditioned stimuli. Together, these findings strongly suggest that tonic β_1_‐adrenergic activity in the auditory cortex of untrained gerbils is involved in the control of mechanisms or brain states that support sound discrimination learning. Interestingly, once FM discrimination learning had occurred (Experiments 1–3), propranolol administration impaired memory retention (depending on an appropriate post‐session time window of injection, as discussed above), but not performance gains during subsequent training. This is consistent with reports on sensory discrimination in rats demonstrating different roles of cortical noradrenaline before and after learning (Brosh, Rosenblum, & Barkai, 2006; Everitt et al., 1983). Potential mechanisms may involve, among others, noradrenergic and/or experience‐dependent alterations in intrinsic and synaptic properties of cortical neurons (Grzelka et al., 2017; Saar & Barkai, 2009). Recently, such mechanisms have been implicated in the modulation of auditory learning and memory in birds (Ross et al., 2019).

Activity of β‐adrenoceptors may recruit transcription‐ and/or translation‐dependent mechanisms to convert synapses into a long‐lasting highly plastic state (Gelinas & Nguyen, 2005; Maity et al., 2015; Mueller, Porter, & Quirk, 2008). Temporary inhibition of protein synthesis in the auditory cortex of untrained gerbils does not influence FM discrimination learning on subsequent days (Kraus et al., 2002; Tischmeyer et al., 2003), implying that the currently monitored late suppressive effect of β‐blockers on FM discrimination learning was induced through processes that were initially translation‐independent. Indeed, noradrenaline may control the modification of proteins and nucleic acids that ultimately participate in the epigenetic regulation of gene expression‐dependent processes in sensory cortices and associated brain regions (Beldjoud, Barsegyan, & Roozendaal, 2015; Hansen, 2017; Oliveira, 2016; Phan & Bieszczad, 2016; Poo et al., 2016). Accordingly, in the rodent auditory cortex and its avian analogue, both noradrenergic and epigenetic mechanisms contribute to information processing that may subserve the encoding of details of behaviourally relevant cues into memory (Bieszczad et al., 2015; Edeline, 2012; Lee, Pawlisch, Macedo‐Lima, & Remage‐Healey, 2018; Phan et al., 2017; Shang, Bylipudi, & Bieszczad, 2019).

According to recent conceptions (Clewett, Huang, Velasco, Lee, & Mather, 2018; Mather et al., 2016), the effects of noradrenergic signalling depend on interactions with local glutamatergic signalling in regions transmitting important information, thus biasing processes at the network level. In the rodent hippocampus, noradrenaline facilitates long‐lasting alterations in synaptic efficacy through mediation of β‐adrenergic and NMDA‐type glutamatergic receptor activities and subsequent engagement of epigenetic mechanisms (Maity, Jarome, Blair, Lubin, & Nguyen, 2016). Even background activity of NMDA receptors modulates synaptic function through epigenetic mechanisms (Chen, Wang, Modrusan, Sheng, & Kaminker, 2014; Nelson, Kavalali, & Monteggia, 2008). Inhibition of β‐adrenoceptors, in turn, may affect background excitatory neurotransmission (Aubert et al., 2001) and induce long‐lasting (>24 hr) changes in synaptic transmission (Kemp & Manahan‐Vaughan, 2008). This suggests that basal synaptic functions are subject to tonic β‐adrenergic control. Supposing similar conditions in the auditory cortex, a temporary inhibition or stimulation of β‐adrenoceptors in naive animals might thus interfere with mechanisms that eventually govern neuronal excitability and network activity during learning.

Furthermore, the expression of GluN2B subunits of cortical NMDA receptors is under monoamine‐modulated epigenetic control (Nghia et al., 2015). Interference with this regulation might thus impact NMDA receptor signalling later, at the time of behavioural training. During auditory discrimination learning, the expression of synaptic GluN2B‐containing NMDA receptors, which have been implicated in the control of gene expression‐dependent forms of synaptic plasticity (Melgarejo da Rosa, Yuanxiang, Brambilla, Kreutz, & Karpova, 2016), is differentially regulated in auditory‐cortical and striatal regions (Kähne et al., 2016; Sun et al., 2005). Increased communication between the auditory cortex and the striatum, a major reinforcement‐analysing structure (Gerraty et al., 2018; Schultz, 2016), enables the transformation of sound representation into goal‐directed motor responses (Schulz et al., 2016; Xiong, Znamenskiy, & Zador, 2015). Encoding of the Go‐response, in turn, critically involves auditory‐cortical NMDA receptor signalling (Schicknick & Tischmeyer, 2006) and was selectively affected in opposite directions when β_1_‐adrenergic antagonists and agonists were previously applied to naive animals in the present study. Given that catecholamines in the brain in general (Birn et al., 2019; van den Brink et al., 2016; Devilbiss, 2019; Guedj, Meunier, Meunier, & Hadj‐Bouziane, 2017; Helbing, Tischmeyer, & Angenstein, 2017) and in the auditory cortex (Happel, 2016; Happel et al., 2014; Reichenbach et al., 2015) shape the functional connectivity of local as well as global circuits, the present findings can be explained by postulating a critical role of β‐adrenergic activity in the auditory cortex for NMDA receptor‐dependent mechanisms of FM discrimination learning that support the strengthening of functional connections required for goal‐directed behaviour.

β‐adrenergic mechanisms in neuronal and glial cells may differentially control components of energy metabolism that are critical during behavioural training and during memory consolidation (Catus, Gibbs, Sato, Summers, & Hutchinson, 2011; Dienel & Cruz, 2016; Gao et al., 2016; Gibbs, Hutchinson, & Summers, 2008; Kong et al., 2017). Additionally, locus coeruleus activity has been implicated in the mobilisation of energy necessary for goal‐directed activity (Bouret & Richmond, 2015). Considering that catecholaminergic activity in the auditory cortex of naive animals (Reichenbach et al., 2015) as well as differential conditioning to FMs (Kähne et al., 2016) cause long‐lasting changes in energy‐related protein profiles and that propranolol strikingly affects energy metabolism in the central auditory system (Savaki, Kadekaro, Jehle, & Sokoloff, 1978), regulation of components of local energy supply, which may also be subject to epigenetic control (O'Byrne et al., 2011), is another conceivable function of auditory‐cortical β‐adrenoceptors that may permit FM discrimination learning.

To summarise, based on the present findings we hypothesise that tonic activity of β_1_‐adrenoceptors in the gerbil auditory cortex may control, among others, epigenetic mechanisms determining the states of synaptic transmission, neural network interaction and/or energy supply required for the acquisition of goal‐directed actions during FM discrimination learning.

### 4.4 Differential effects of propranolol on FM detection and discrimination learning

Contrary to the inhibitory effect on FM discrimination learning, active avoidance learning with a single FM sweep was not impaired after auditory‐cortical propranolol injection (Experiment 8). Likewise, post‐acquisition propranolol infusion in Experiment 1, though suppressing the discrimination increment normally occurring between sessions 1 and 2, did not affect the increment in the total number of hurdle crossings in response to sweep presentation per se. These findings indicate that the impact of locally applied β‐blockers on FM discrimination does not reflect effects on sensory and motor systems and associative mechanisms required for CS detection and for acquisition, consolidation and expression of the hurdle response to avoid foot shock. Moreover, given that central noradrenergic lesion (Radwanska, Nikolaev, & Kaczmarek, 2010) or intrahippocampally infused propranolol (Lv et al., 2017) inhibits single‐sound active avoidance learning in rats, normal active avoidance learning found in the present study after auditory‐cortical propranolol infusion implies that the suppressive effect on FM discrimination learning does not reflect drug actions in adjacent brain regions.

Differential conditioning to sounds in a shuttle‐box initially causes an association between the presentation of the sounds and the likelihood of foot shocks as reinforcers. With an increasing chance to avoid foot shocks by crossing the hurdle during CS+ presentation and by suppressing this response during CS‐ presentation, these meaningful associations are formed and must be consolidated and recalled in order to select appropriate response strategies. Detection and discrimination of behaviourally relevant sounds represent hierarchical learning steps supposed to induce distinct forms of retuning of auditory cortex neurons (Ohl, 2015; Scheich et al., 2011), that is, best frequency shift and slope sharpening of frequency receptive field tuning, respectively. The different types of learning‐relevant plasticity observed in the auditory cortex may be controlled, among others, by epigenetic mechanisms (Bieszczad et al., 2015) and by noradrenaline (Edeline et al., 2011), which may modulate the retuning of individual auditory cortex neurons differentially through mediation of α_1_‐, α_2_‐ and β‐adrenoceptor activities (Gaucher & Edeline, 2015; Manunta & Edeline, 1997, 2004). Given that the auditory cortex participates in associative learning mediated through both detection as well as discrimination of FM sweeps (Letzkus et al., 2011; Ohl et al., 1999), the differential effects of propranolol monitored in the present study suggest that β‐adrenergic mechanisms in the auditory cortex bear task‐dependent importance for auditory learning and/or for consolidating the different types of learning‐induced representational plasticity that may occur depending on the type of the task.

Active avoidance conditioning and differential conditioning to FMs in the present study were performed in the same shuttle‐box by use of the same Go‐stimulus and the same electrodermal stimulus, so that reinforcement quality and procedural aspects were largely identical. The task‐specific effect of β‐adrenoceptor inhibition should therefore predominantly reflect actions on the more complex sensory and cognitive operations during FM discrimination learning, such as distinguishing the directions of the sweeps and forming meaningful associations, which ultimately permit the selection of appropriate response strategies. Processing of information about distinctive features of FMs and acquisition of goal‐directed action critically depend on the auditory cortex (Ohl et al., 1999). Studies in guinea pigs revealed enhancing effects of noradrenaline on the ability of subpopulations of auditory cortex neurons to discriminate between complex natural sounds (Gaucher & Edeline, 2015). Supposing similar conditions in the gerbil, the task‐specific suppressive effect of β‐blockers on FM discrimination learning monitored in the present study strongly suggests a critical role of tonic β‐adrenoceptor activity even under basal conditions for maintaining an adequate subset of auditory cortex neurons in a state appropriate to distinguish a common feature of natural sounds, namely frequency modulations.

**Figure 9:**
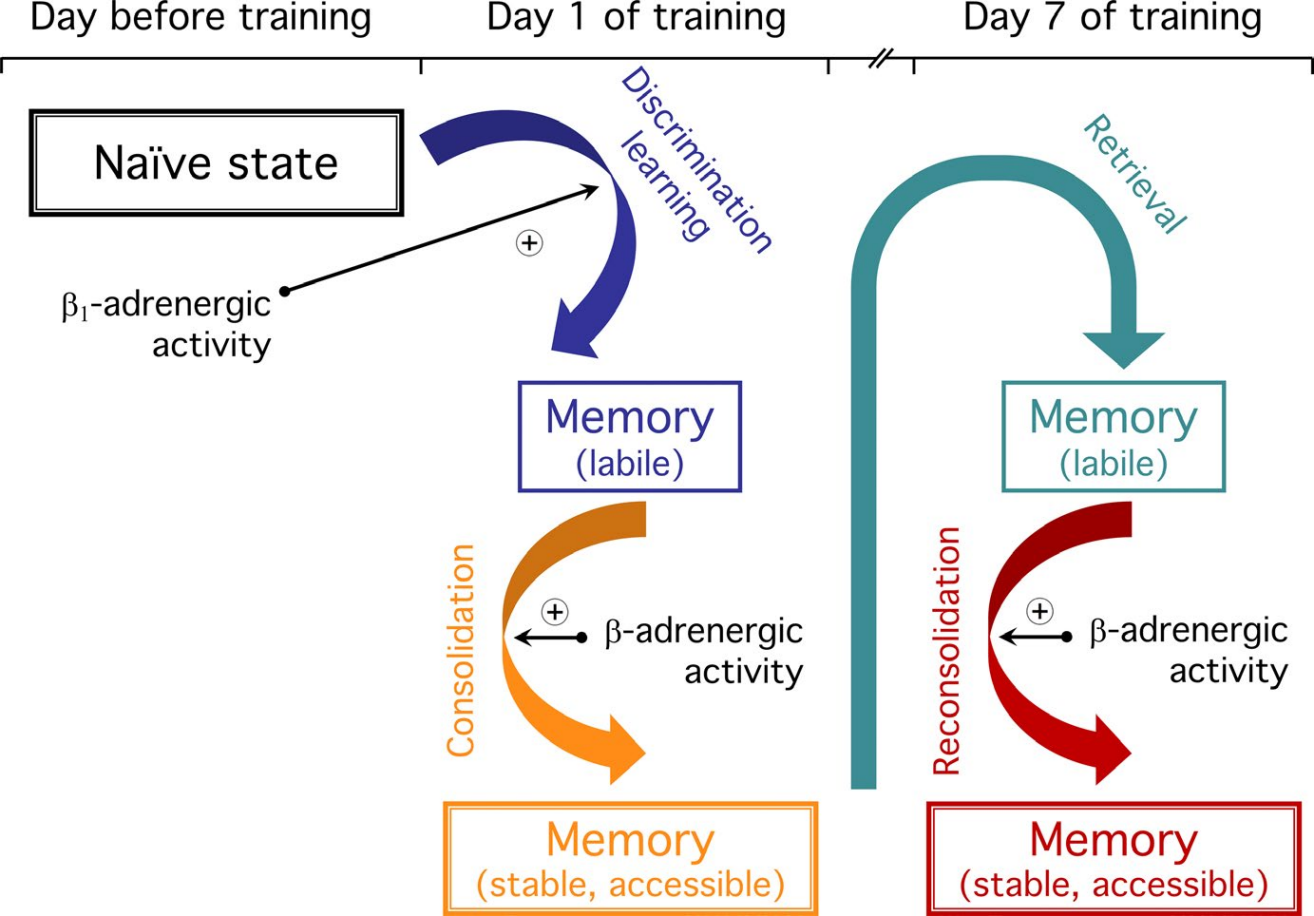
Various effects of β‐adrenergic modulation of FM discrimination learning and memory in the auditory cortex. Based on our data, we propose a hypothetical model for β‐adrenoceptor signalling during processes of memory formation, consolidation and reconsolidation. First, in naive animals, that is, without explicit task engagement, β_1_‐adrenoceptor activity promotes mechanisms that facilitate subsequent FM discrimination (not detection) learning. Second, after learning, the retention and/or proper retrieval of newly acquired memory critically depend on the β‐adrenergic modulation of long‐term memory consolidation. Third, the reconsolidation of previously established FM discrimination memory after retrieval in a retraining session, though relying on mechanisms that may differ from those recruited during the consolidation of newly acquired memory, also requires β‐adrenergic activity in the auditory cortex during a narrow post‐session time window. A more comprehensive model of modulatory processes of memory management by the auditory cortex is presented in Figure S12. [Colour figure can be viewed at http://wileyonlinelibrary.com]

Altogether, the present findings suggest that β‐adrenergic receptor signalling in the auditory cortex controls mechanisms that permit FM discrimination learning and support memory retention and/or retrieval after learning and rehearsal (Figure 9).

### 4.5 Catecholaminergic modulation of FM discrimination learning and memory

Considering also previous studies on this paradigm, the current view of FM‐conditioned memory formation and its catecholaminergic modulation in the auditory cortex is sketched in Figure S12. According to this simplified model, differential conditioning to FMs induces increased cortical neurotransmitter release, activation of NMDA‐type glutamate receptors, mTOR signalling and translation‐dependent changes. These processes precede alterations in non‐synaptic and synaptic proteomes of cortical, hippocampal and striatal networks, probably reflecting memory‐relevant plastic rearrangements. Within this framework of neural plasticity, catecholaminergic systems in the auditory cortex are thought to fulfil complementary, overlapping and/or independent modulatory functions, depending in part on the learning history: in untrained animals, activities of β_1_‐adrenoceptors and D_1/5_‐dopamine receptors set the stage for future events of sound discrimination learning and subsequent long‐term memory formation, respectively; once discrimination learning has occurred, supportive functions of noradrenergic and dopaminergic systems temporarily converge on post‐session memory fixation.

## CONFLICT OF INTERESTS

The authors declare no competing financial interests.

## AUTHOR CONTRIBUTIONS

WT, FWO, EB and EDG designed the study. HS and JH performed the experiments. WT, HS and JH analysed the data. WT wrote the manuscript. All of the authors discussed the results and finalised the manuscript.

## Data Availability

The data are available on request to the corresponding author for researchers who meet the criteria for access to confidential data without limitations.
