# Supplementary material for: β‐adrenergic modulation of discrimination learning and memory in the auditory cortex
Source: Eur J Neurosci. 2019 Jul 1;50(7):3141–63. doi: 10.1111/ejn.14480 (PMC6900137; doi:10.1111/ejn.14480)
Supplement: Supplementary file 1 [file EJN-50-3141-s001.pdf]

Supporting Information for:

β-adrenergic modulation of discrimination learning and memory in the auditory cortex

(Schicknick *et al.*)

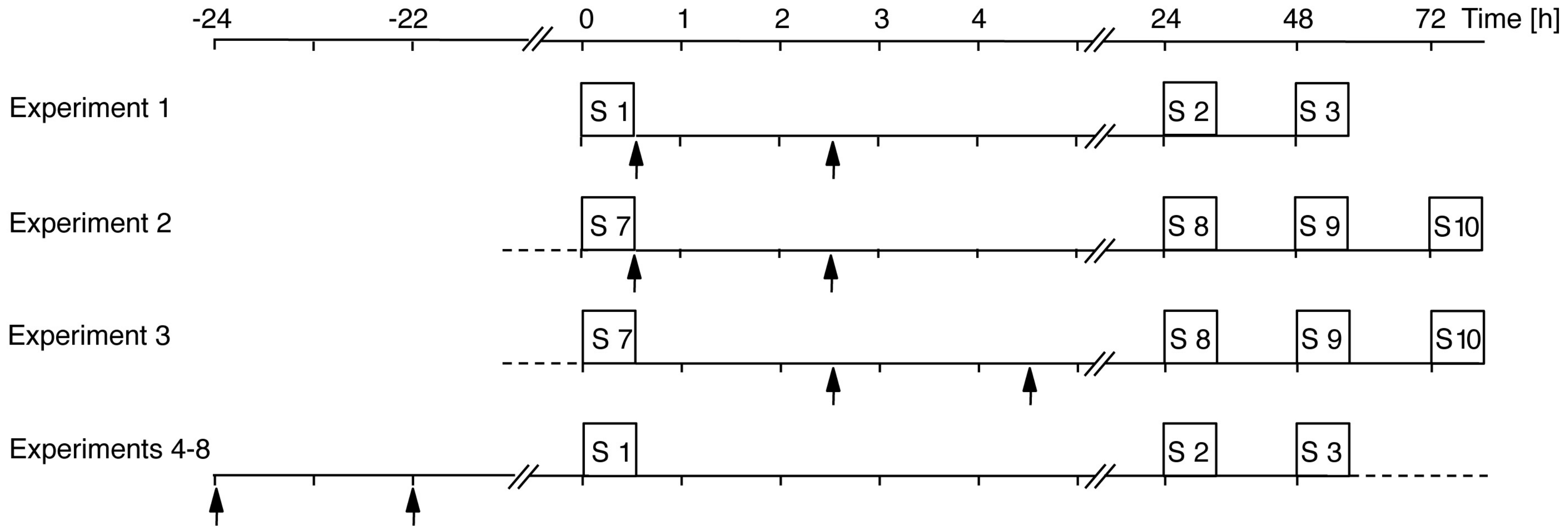

Supplementary Figure S1. Scheme of pharmacological treatments. Injections (arrows) of pharmacological agents or vehicle were applied bilaterally to the auditory cortex at the indicated time intervals in relation to training session 1 or training session 7 (session-numbers in boxes).

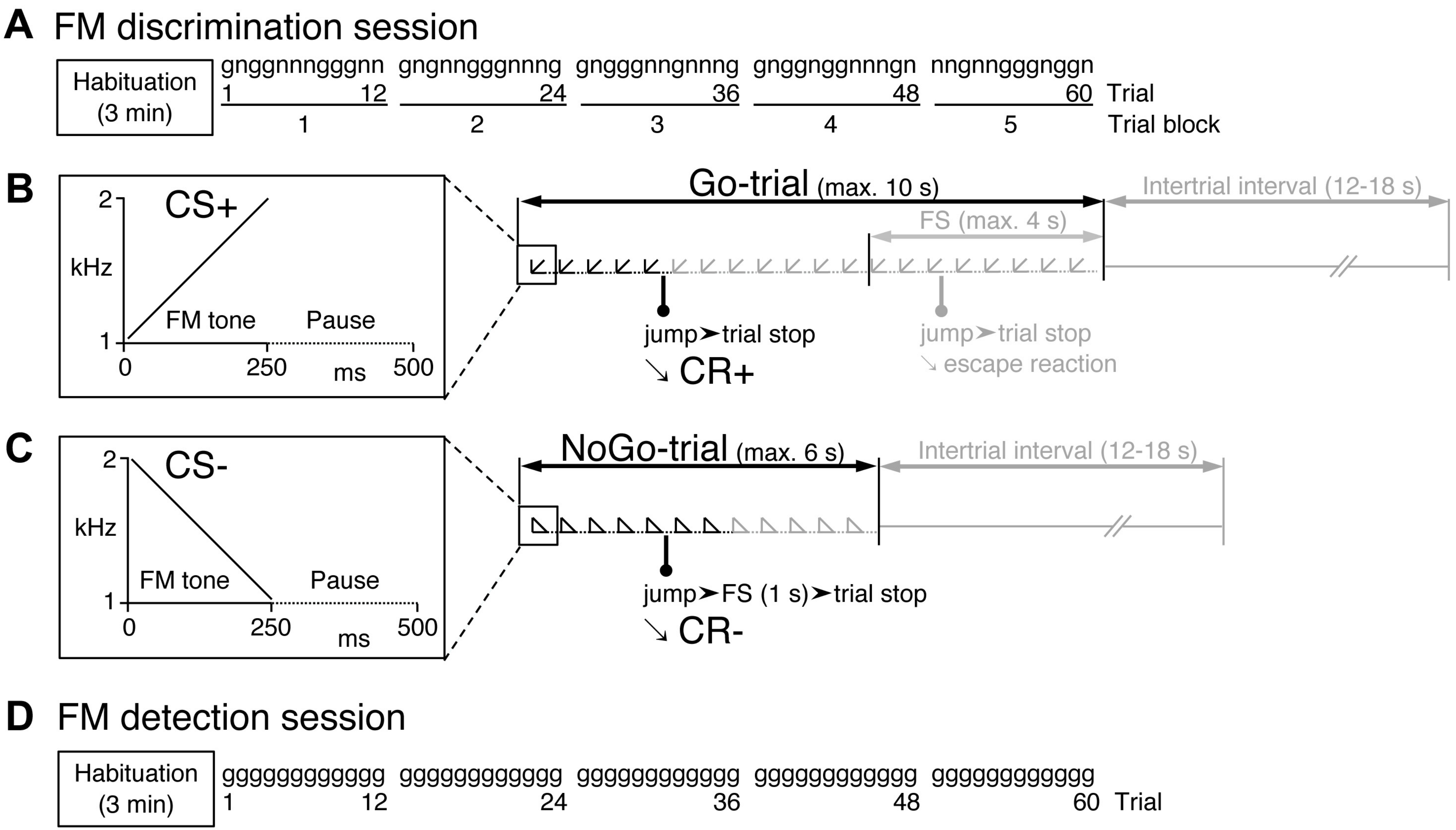

Supplementary Figure S2. Design of behavioural experiments. (A) Structure of a FM discrimination training session; 'g' stands for a Go-trial, 'n' stands for a NoGo-trial. (B) CS+ and Go-trial parameters (FS, foot-shock). (C) CS- and NoGo-trial parameters. (D) Structure of a FM detection training session; 'g' stands for a trial with parameters identical to those of Go-trials shown in B.

Methodological considerations

In this study, a broad set of behavioural data was collected to control for unspecific actions of pharmacological treatments that require attention. (1) Procedures required for local drug delivery, like surgery and anaesthesia, and intracortical saline injections caused no significant impairments in FM discrimination compared to the pre-treatment level and to the level of gerbils with no surgery, anaesthesia or injection (Supplementary Figure S3). This is consistent with our previous findings on gerbils subjected to intracortical saline injections (see*, e.g.*, page 673 in Schicknick, H. & Tischmeyer, W. 2006, *Neuropharmacology*, **50**, 671-676, and Figure 8A in Schicknick, H. *et al.* 2012, *Eur J Neurosci*, **35**, 763-774).
(2) General parameters recorded during the behavioural experiments (Supplementary Figures S4-S11) suggest that the infused drugs caused no alterations in the states of arousal and activity or deficits in sensory transduction and motor systems that could account for performance changes one day post-injection. (3) Post-acquisition propranolol-treated gerbils increased their discrimination rates normally within the following training session (Figure 1), indicating that one day after local injection into the auditory cortex neither β-adrenoceptor antagonism nor potential adrenoceptor-independent actions of propranolol and its metabolites affected mechanisms required for sensory processing of FMs, improving their discrimination, and sensorimotor integration necessary to express the discriminative behaviour. (4) Suppressive effects of post-session propranolol treatment depended on a narrow temporal relation to the behavioural experiment (Figure 2) and may thus not reflect a sustained presence or toxic side effect of the antagonist. This agrees with studies in rats demonstrating that intracerebrally administered propranolol is eliminated with a half-life of ≈2 h (Smits, J.M. & Struyker-Boudier, H.A. 1979, *Naunyn Schmiedebergs Arch Pharmacol*, **309**, 19-24) and does not cause lingering damage (Bahar, A. *et al.* 2003, *Eur J Neurosci*, **17**, 1527-1530). (5) β-adrenergic antagonists and agonists influenced FM discrimination learning in opposite directions, thus providing support regarding the specificity of the effects. (6) Pharmacological treatments affected the behavioural responses to CS+ and CS- differentially, suggesting that the effects were not caused due to drug actions on general mechanisms that may interfere with FM discrimination learning and performance, such as CS detection and/or response initiation. Accordingly, propranolol did not affect FM detection learning (Figure 7).

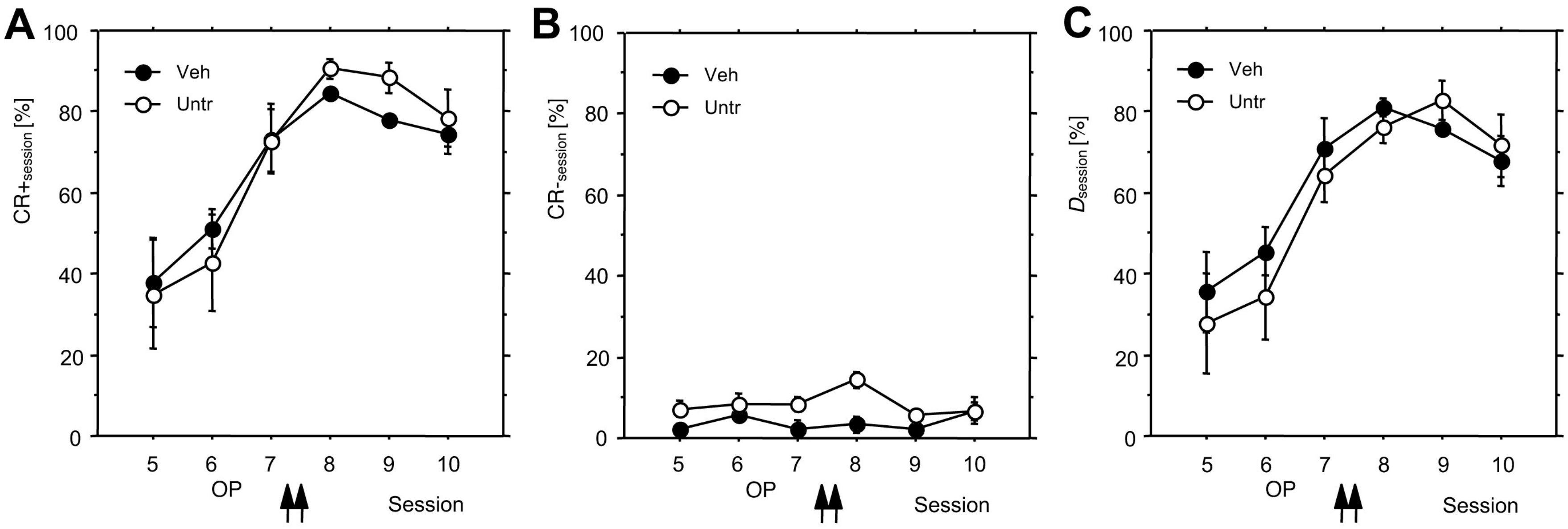

Supplementary Figure S3. Procedures required for local drug delivery cause no impairments in FM discrimination learning and performance. Gerbils were trained on the FM discrimination for 10 sessions. Vehicle-treated gerbils (Veh, *n*=3) were subjected to surgical operation (OP) under ketamine-xylazine anaesthesia after session 6 and to saline injections into the auditory cortex under light halothane anaesthesia twice, *i.e.,* 2 and 4 h after session 7 (indicated by arrows). Surgical procedures and intracortical injections were performed essentially as described in the Methods section of the manuscript. Untreated gerbils (Untr, *n*=6) were not subjected to surgery, anaesthesia, and injections. (A) Relative frequencies of correct conditioned responses ($CR+_{session}$). (B) Relative frequencies of false alarms ($CR-_{session}$). (C) Discrimination rates ($D_{session}$, *i.e.*, the differences between $CR+_{session}$ and $CR-_{session}$). Data are expressed as group means per training session ±SEM. Values of RM-ANOVA comparing behavioural measures over sessions 6-10 across treatment groups: (A) treatment effect, $F_{(1,7)}$=0.13, P=0.733; session effect, $F_{(4,28)}$=9.26, P<0.0001; treatment x session, $F_{(4,28)}$=0.48, P=0.752; (B) treatment effect, $F_{(1,7)}$=5.36, P=0.054; session effect, $F_{(4,28)}$=1.48, P=0.235; treatment x session, $F_{(4,28)}$=1.85, P=0.146; (C) treatment effect, $F_{(1,7)}$=0.11, P=0.746; session effect, $F_{(4,28)}$=11.43, P<0.0001; treatment x session, $F_{(4,28)}$=0.65, P=0.629.

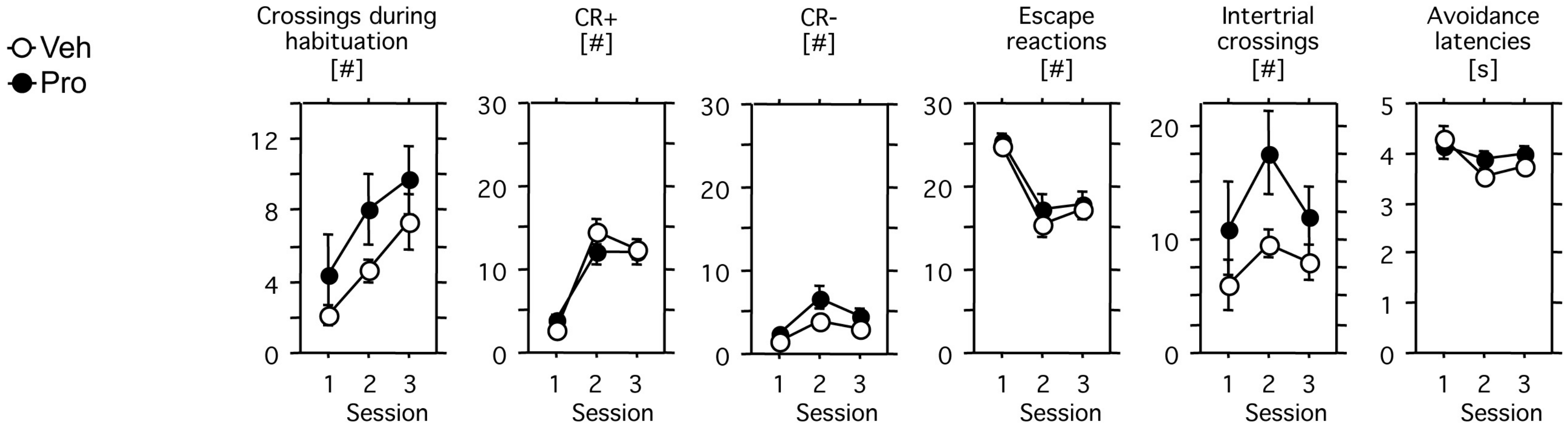

RM-ANOVA		Crossings during habituation	CR+	CR-	Escape reactions	Intertrial crossings	Avoidance latencies
Treatment effect	$F_{(1,28)}$	2.204	0.110	4.088	0.816	3.982	0.815
	P	0.1488	0.7430	0.0528	0.3739	0.0558	0.3743
Session effect	$F_{(2,56)}$	9.879	51,435	14.266	34.571	2.843	5.794
	P	**0.0002**	**<0.0001**	**<0.0001**	**<0.0001**	0.0667	**0.0052**
Treatment x session	$F_{(2,56)}$	0.136	1.218	1.299	0.228	0.427	1.845
	P	0.8735	0.3035	0.2810	0.7971	0.6549	0.1675

Supplementary Figure S4. Behavioural data collected in Experiment 1. Data were recorded during 3 min of habituation to the shuttle-box and during FM discrimination training of gerbils infused with vehicle (Veh, *n*=15) or 33.8 mM propranolol (Pro, *n*=15) into the auditory cortex twice, immediately and 2 h after completion of session 1. Gerbils were exposed to the shuttle-box for 3 min immediately before each training session to adapt to the experimental environment. During these habituation periods, the numbers of hurdle crossings were recorded. During the subsequent FM discrimination training, correct conditioned reactions (CR+), false alarms (CR-), escape reactions, intertrial crossings, and avoidance latencies (*i.e.,* the average time to initiate CR+) were monitored. Upper part: experimental data expressed as group means ±SEM. Lower part: values of RM-ANOVA comparing behavioural measures over sessions 1-3 across pharmacological treatment conditions. Significant values (*P*<0.05) in bold.

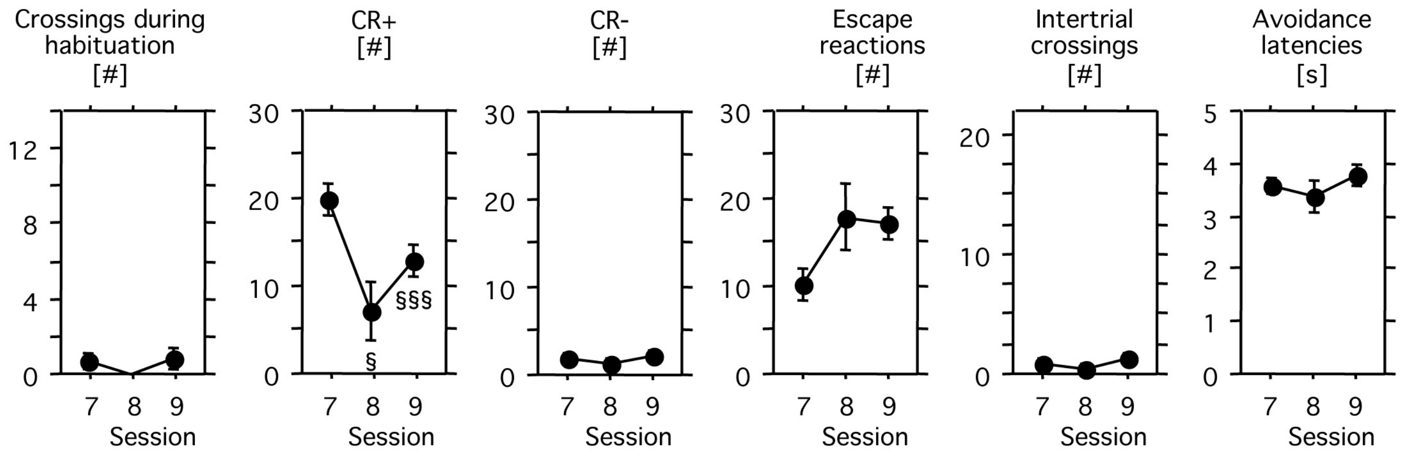

RM-ANOVA Session effect		Crossings during habituation	CR+	CR-	Escape reactions	Intertrial crossings	Avoidance latencies
	$F_{(2,10)}$	2,333	6.653	0.636	3.433	1.145	0.246
	P	0.1473	**0.0145**	0.5494	0.0733	0.3568	0.7863

Supplementary Figure S5. Behavioural data collected in Experiment 2. Data were recorded during 3 min of habituation to the shuttle-box and during FM discrimination training of gerbils infused with 33.8 mM propranolol (n=6) into the auditory cortex twice, immediately and 2 h after completion of session 7. Gerbils were exposed to the shuttle-box for 3 min immediately before each training session to adapt to the experimental environment. During these habituation periods, the numbers of hurdle crossings were recorded. During the subsequent FM discrimination training, correct conditioned reactions (CR+), false alarms (CR-), escape reactions, intertrial crossings, and avoidance latencies (*i.e.,* the average time to initiate CR+) were monitored. Upper part: experimental data expressed as group means ±SEM. $^{§}P<0.05$, $^{§§§}P<0.005$, significantly different from the value in session 7 (paired t-test). Lower part: values of RM-ANOVA comparing behavioural measures over sessions 7-9. Significant values ($P<0.05$) in bold.

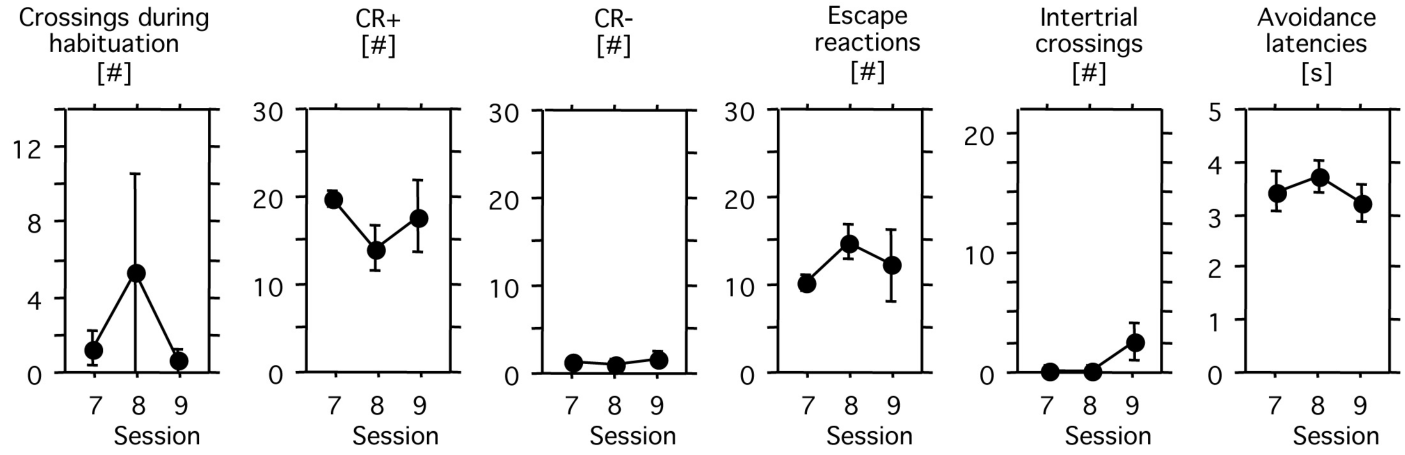

RM-ANOVA Session effect	$F_{(2,4)}$	0.905	2.104	0.286	1.138	1.960	1.259
	P	0.4739	0.2375	0.7656	0.4061	0.2551	0.3766

Supplementary Figure S6. Behavioural data collected in Experiment 3. Data were recorded during 3 min of habituation to the shuttle-box and during FM discrimination training of gerbils infused with 33.8 mM propranolol (*n*=3) into the auditory cortex twice, 2 h and 4 h after completion of session 7. Gerbils were exposed to the shuttle-box for 3 min immediately before each training session to adapt to the experimental environment. During these habituation periods, the numbers of hurdle crossings were recorded. During the subsequent FM discrimination training, correct conditioned reactions (CR+), false alarms (CR-), escape reactions, intertrial crossings, and avoidance latencies (*i.e.,* the average time to initiate CR+) were monitored. Upper part: experimental data expressed as group means ±SEM. Lower part: values of RM-ANOVA comparing behavioural measures over sessions 7-9.

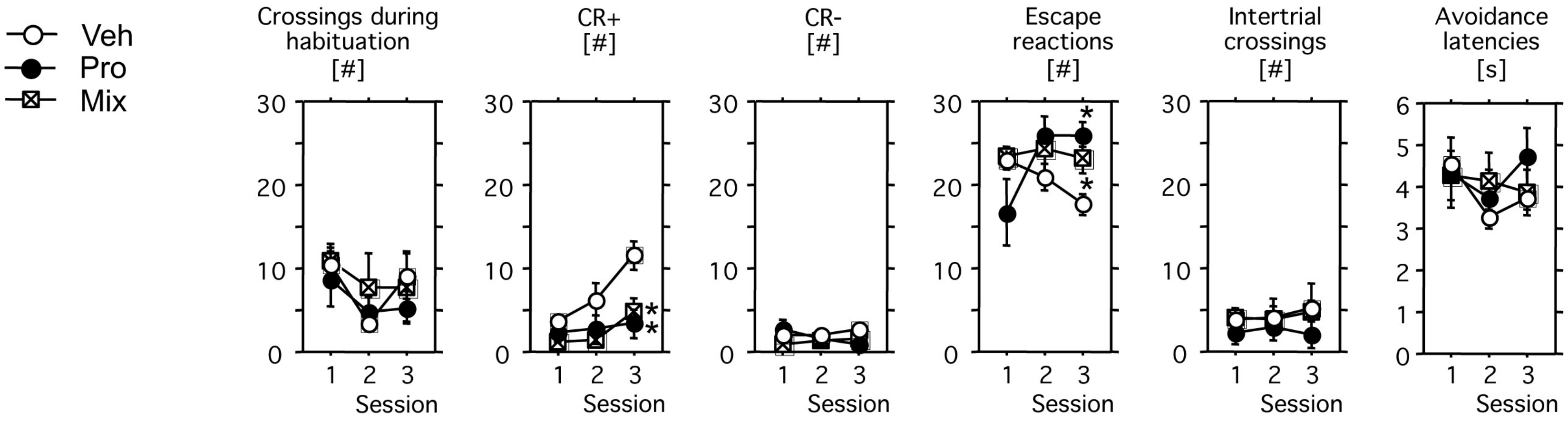

RM-ANOVA		Crossings during habituation	CR+	CR-	Escape reactions	Intertrial crossings	Avoidance latencies
Treatment effect	$F_{(2,14)}$	0.317	9.839	1.197	3.005	0.382	0.696
	P	0.7333	**0.0021**	0.3313	0.0821	0.6895	0.5152
Session effect	$F_{(2,28)}$	2.050	5.548	0.073	1.712	0.068	1.023
	P	0.1476	**0.0093**	0.9297	0.1989	0.9343	0.3727
Treatment x session	$F_{(4,28)}$	0.427	1.392	0.747	4.187	0.095	0.579
	P	0.7881	0.2623	0.5680	**0.0088**	0.9831	0.6801

Supplementary Figure S7. Behavioural data collected in Experiment 4. Data were recorded during 3 min of habituation to the shuttle-box and during FM discrimination training of gerbils infused with vehicle (Veh, *n*=8), 33.8 mM propranolol (Pro, *n*=3) or a mixture (Mix, *n*=6) of 3.8 mM atenolol and 0.32 µM ICI118,551 into the auditory cortex twice, 24 h and 22 h before the start of session 1. Gerbils were exposed to the shuttle-box for 3 min immediately before each training session to adapt to the experimental environment. During these habituation periods, the numbers of hurdle crossings were recorded. During the subsequent FM discrimination training, correct conditioned reactions (CR+), false alarms (CR-), escape reactions, intertrial crossings, and avoidance latencies (*i.e.,* the average time to initiate CR+) were monitored. Upper part: experimental data expressed as group means ±SEM. **P*<0.05, significantly different from Veh (Dunnett's test). Lower part: values of RM-ANOVA comparing behavioural measures over sessions 1-3 across pharmacological treatment conditions. Significant values (*P*<0.05) in bold.

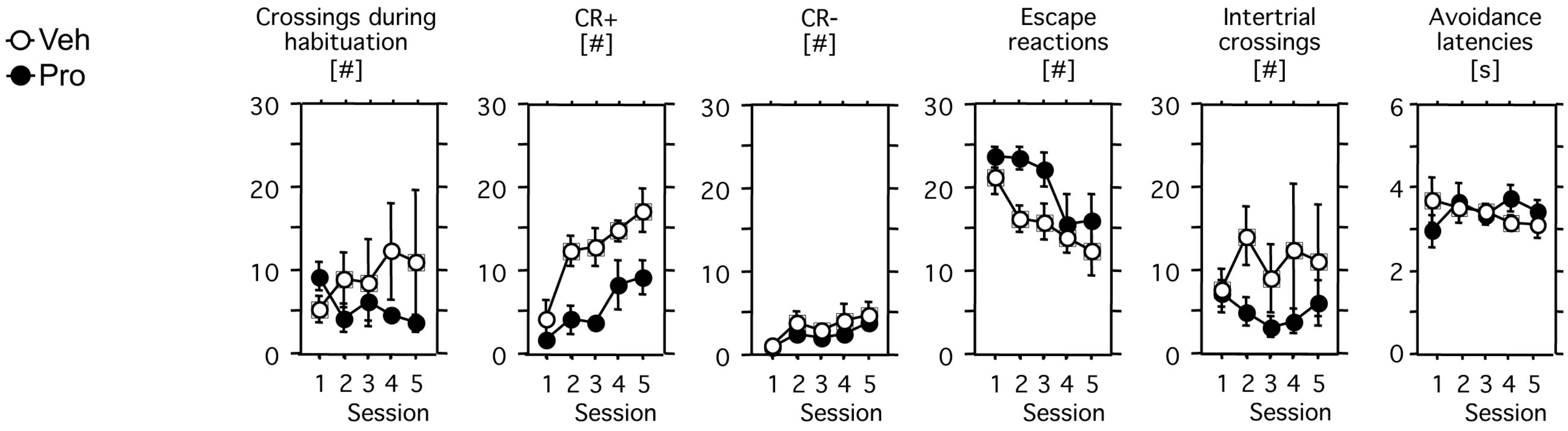

RM-ANOVA		Crossings during habituation	CR+	CR-	Escape reactions	Intertrial crossings	Avoidance latencies
Treatment effect	$F_{(1,9)}$	0.799	24.891	2.711	5.210	2.319	2.220
	P	0.3946	**0.0008**	0.1341	**0.0484**	0.1621	0.1704
Session effect	$F_{(4,36)}$	0.129	8.461	3.161	5.700	0.398	0.449
	P	0.9710	**<0.0001**	**0.0252**	**0.0012**	0.8088	0.7725
Treatment x session	$F_{(4,36)}$	1.662	0.932	0.195	0.707	0.689	0.640
	P	0.1803	0.4563	0.9393	0.5923	0.6041	0.6371

Supplementary Figure S8. Behavioural data collected in Experiment 5. Data were recorded during 3 min of habituation to the shuttle-box and during FM discrimination training of gerbils infused with vehicle (Veh, *n*=5) or 33.8 mM propranolol (Pro, *n*=6) into the auditory cortex twice, 24 h and 22 h before the start of session 1. Gerbils were exposed to the shuttle-box for 3 min immediately before each training session to adapt to the experimental environment. During these habituation periods, the numbers of hurdle crossings were recorded. During the subsequent FM discrimination training, correct conditioned reactions (CR+), false alarms (CR-), escape reactions, intertrial crossings, and avoidance latencies (*i.e.,* the average time to initiate CR+) were monitored. Upper part: experimental data expressed as group means ±SEM. Lower part: values of RM-ANOVA comparing behavioural measures over sessions 1-5 across pharmacological treatment conditions. Significant values ($P<0.05$) in bold.

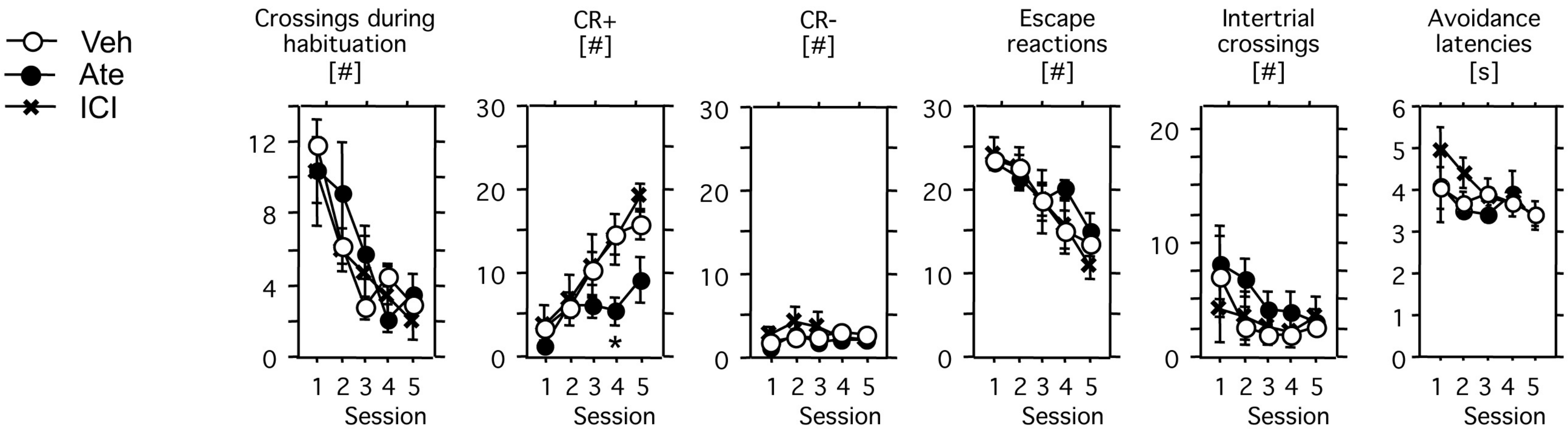

RM-ANOVA		Crossings during habituation	CR+	CR-	Escape reactions	Intertrial crossings	Avoidance latencies
Treatment effect	$F_{(2,15)}$	0.351	2.924	1.338	0.222	0.942	0.842
	P	0.7098	0.0847	0.2919	0.8034	0.4116	0.4502
Session effect	$F_{(4,60)}$	14.985	25.537	1.228	18.666	1.820	2.267
	P	**<0.0001**	**<0.0001**	0.3085	**<0.0001**	0.1367	0.0724
Treatment x session	$F_{(8,60)}$	1.086	2.539	0.595	0.999	0.317	0.467
	P	0.3851	**0.0189**	0.7779	0.4462	0.9565	0.8744

Supplementary Figure S9. Behavioural data collected in Experiment 6. Data were recorded during 3 min of habituation to the shuttle-box and during FM discrimination training of gerbils infused with vehicle (Veh, *n*=9), 3.8 mM atenolol (Ate, *n*=6), or 0.32 µM ICI118,551 (ICI, *n*=3) into the auditory cortex twice, 24 h and 22 h before the start of session 1. Gerbils were exposed to the shuttle-box for 3 min immediately before each training session to adapt to the experimental environment. During these habituation periods, the numbers of hurdle crossings were recorded. During the subsequent FM discrimination training, correct conditioned reactions (CR+), false alarms (CR-), escape reactions, intertrial crossings, and avoidance latencies (*i.e.,* the average time to initiate CR+) were monitored. Upper part: experimental data expressed as group means ±SEM. *P<0.05, significantly different from Veh (Dunnett's test). Lower part: values of RM-ANOVA comparing behavioural measures over sessions 1-5 across pharmacological treatment conditions. Significant values (P<0.05) in bold.

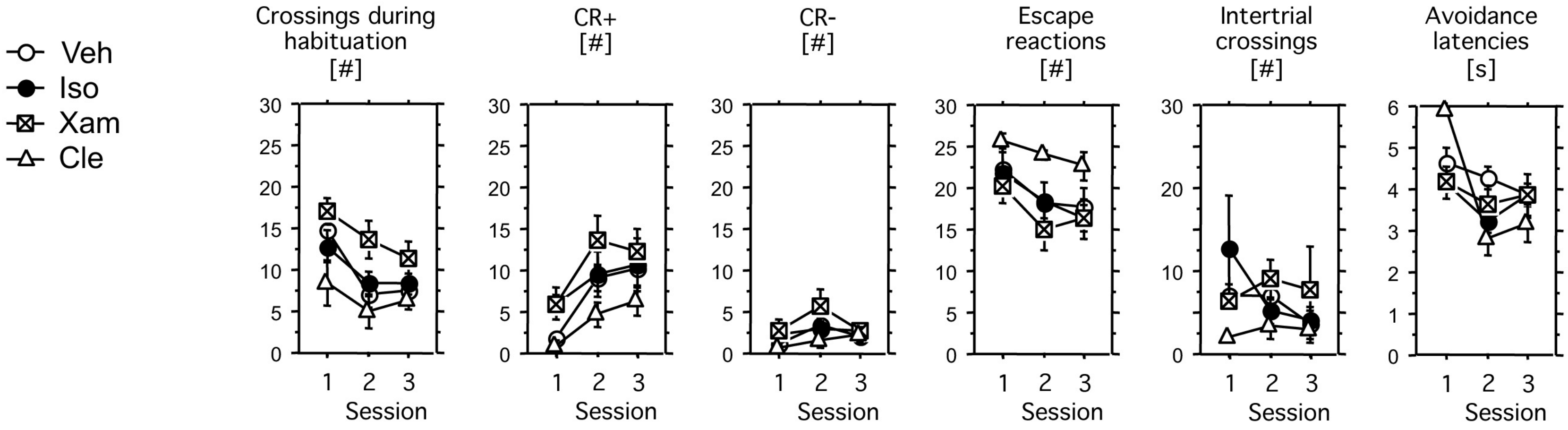

RM-ANOVA		Crossings during habituation	CR+	CR-	Escape reactions	Intertrial crossings	Avoidance latencies
Treatment effect	$F_{(3,18)}$	2.191	1.463	0.917	2.216	0.795	1.402
	P	0.1243	0.2581	0.4524	0.1213	0.5128	0.2746
Session effect	$F_{(2,36)}$	8.821	13,689	6.394	2.847	0.838	8.180
	P	**0.0008**	**<0.0001**	**0.0042**	0.0711	0.4407	**0.0012**
Treatment x session	$F_{(6,36)}$	0.653	0.604	1.125	0.112	1.116	2.120
	P	0.6878	0.7256	0.3677	0.9944	0.3725	0.0749

Supplementary Figure S10. Behavioural data collected in Experiment 7. Data were recorded during 3 min of habituation to the shuttle-box and during FM discrimination training of gerbils infused with vehicle (Veh, n=10), 2.8 mM isoproterenol (Iso, n=6), 10 mM Xamoterol (Xam, n=3), or 0.32 mM clenbuterol (Cle, n=3) into the auditory cortex twice, 24 h and 22 h before the start of session 1. Gerbils were exposed to the shuttle-box for 3 min immediately before each training session to adapt to the experimental environment. During these habituation periods, the numbers of hurdle crossings were recorded. During the subsequent FM discrimination training, correct conditioned reactions (CR+), false alarms (CR-), escape reactions, intertrial crossings, and avoidance latencies (*i.e.,* the average time to initiate CR+) were monitored. Upper part: experimental data expressed as group means ±SEM. Lower part: values of RM-ANOVA comparing behavioural measures over sessions 1-3 across pharmacological treatment conditions. Significant values (P<0.05) in bold.

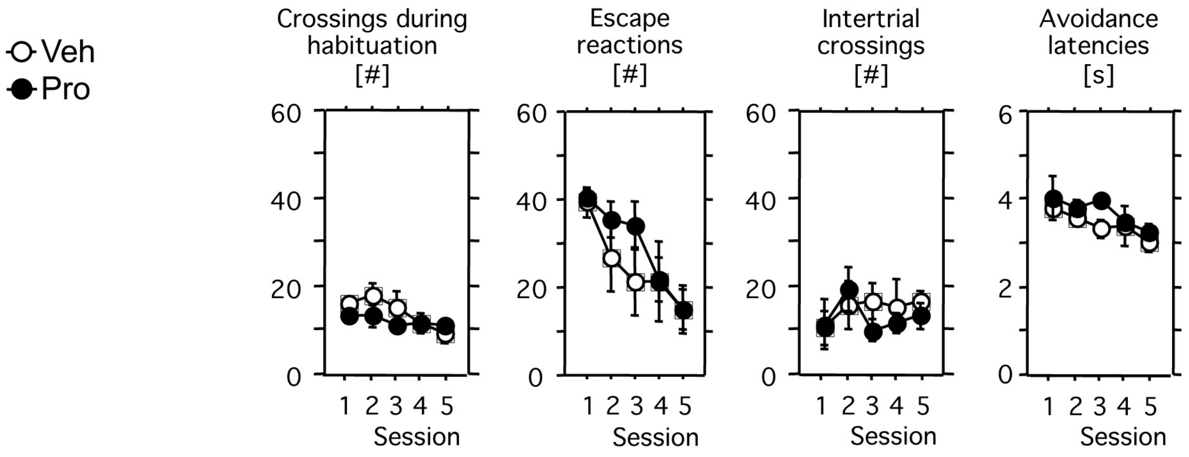

RM-ANOVA		Crossings during habituation	Escape reactions	Intertrial crossings	Avoidance latencies
Treatment effect	$F_{(1,10)}$	1.083	0.581	0.206	1.297
	P	0.3225	0.4637	0.6593	0.2812
Session effect	$F_{(4,40)}$	2.670	9.930	0.953	3.094
	P	**0.0459**	**<0.0001**	0.4438	**0.0261**
Treatment x session	$F_{(4,40)}$	1.047	0.935	0.663	0.383
	P	0.3951	0.4533	0.6214	0.8196

Supplementary Figure S11. Behavioural data collected in Experiment 8. Data were recorded during 3 min of habituation to the shuttle-box and during FM detection training of gerbils infused with vehicle (Veh, n=6) or 33.8 mM propranolol (Pro, n=6) into the auditory cortex twice, 24 h and 22 h before the start of session 1. Gerbils were exposed to the shuttle-box for 3 min immediately before each training session to adapt to the experimental environment. During these habituation periods, the numbers of hurdle crossings were recorded. During the subsequent FM detection training, escape reactions, intertrial crossings, and avoidance latencies were monitored. Upper part: experimental data expressed as group means ±SEM. Lower part: values of RM-ANOVA comparing behavioural measures over training sessions 1-5 across pharmacological treatment conditions. Significant values (P<0.05) in bold.

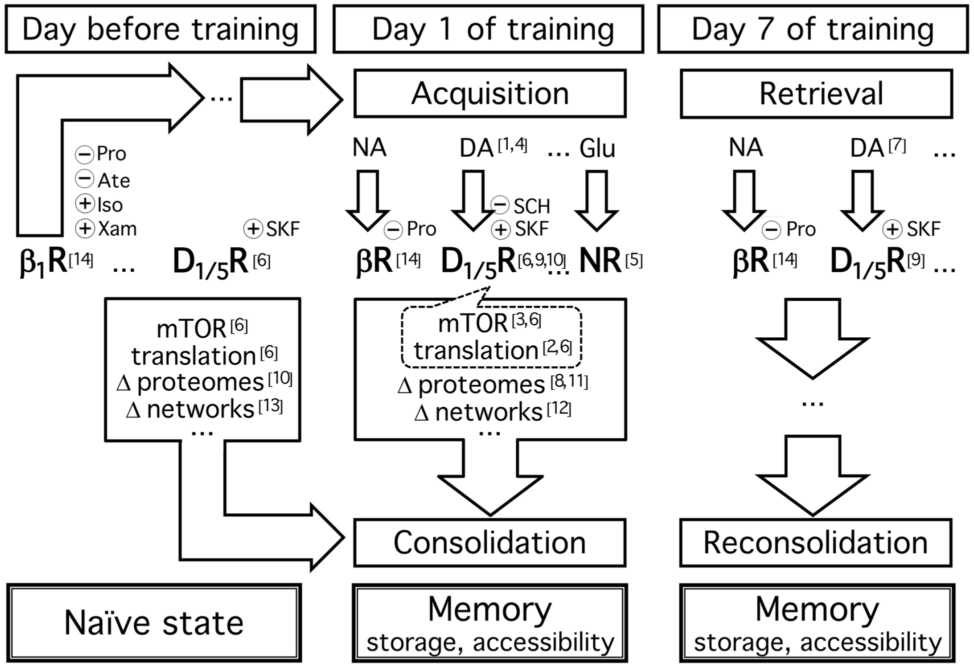

Supplementary Figure S12. Simplified model of FM discrimination learning and memory formation and its catecholaminergic modulation in the auditory cortex. FM discrimination training is thought to induce the cortical release of neuromodulators and neurotransmitters, including noradrenaline, dopamine and glutamate. Pharmacological studies revealed consolidation processes during a narrow post-session time window, which are critical for the retention of the newly acquired memory and for its proper retrieval. These processes require the activation of β-adrenoceptors, $D_{1/5}$-dopamine receptors, and NMDA-type glutamate receptors, as well as the recruitment of mTOR-mediated, protein synthesis-dependent mechanisms. The latter are modulated at least in part through $D_{1/5}$-dopamine receptor signalling. Subsequent changes in non-synaptic and synaptic proteomes of cortical and subcortical brain regions and alterations in their interaction supposedly reflect memory-relevant plastic rearrangements. The reconsolidation of previously established FM discrimination memory after retrieval in a retraining session, although relying on mechanisms that may differ from those recruited during the consolidation of newly acquired memory, is also concordantly supported by post-session β-adrenergic and $D_{1/5}$-dopaminergic activity in the auditory cortex. In contrast, the effects of pharmacological interference applied either without behavioural training or one day prior to the first training session point to differential modulatory functions of catecholaminergic systems in naive animals, suggesting that β_1-adrenoceptor signalling supports mechanisms recruited during the initial acquisition of the discrimination while $D_{1/5}$-dopamine receptor activity preferentially supports mechanisms involved in long-term memory consolidation. "+", process enhancement; "-", process impairment; "...", additional, unknown pathways. Abbreviations: Ate, atenolol; βR, β-adrenoceptor; β_1R, β_1-adrenoceptor; DA, dopamine; $D_{1/5}$R, $D_{1/5}$-dopamine receptor; Glu, glutamate; Iso, isoproterenol; NA, noradrenaline; NR, NMDA-type glutamate receptor; Pro, propranolol; SCH, SCH23390 ($D_{1/5}$R antagonist); SKF, SKF38393, SKF83959 or SKF83822 ($D_{1/5}$R agonists); Xam, xamoterol. References: [1](Stark & Scheich, 1997), [2](Kraus *et al.*, 2002), [3](Tischmeyer *et al.*, 2003), [4](Stark *et al.*, 2004), [5](Schicknick & Tischmeyer, 2006), [6](Schicknick *et al.*, 2008), [7](Rothe *et al.*, 2009), [8](Kähne *et al.*, 2012), [9](Schicknick *et al.*, 2012), [10](Reichenbach *et al.*, 2015), [11](Kähne *et al.*, 2016), [12](Schulz *et al.*, 2016), [13](Helbing *et al.*, 2017), [14](present study).
